# Anti-diabetic medications to fight Parkinson's disease and dementia with Lewy bodies: a pilot study

**DOI:** 10.3389/fneur.2026.1825229

**Published:** 2026-05-29

**Authors:** Tomasz Chmiela, Jarosław Dulski, Michael G. Heckman, Zhihui J. Fang, Zbigniew K. Wszolek, Jessica R. Wilson

**Affiliations:** 1 Department of Neurology, Mayo Clinic, Jacksonville, FL, Unites States; 2 Department of Neurology, Faculty of Medical Sciences, Medical University of Silesia, Katowice, Poland; 3 Division of Neurological and Psychiatric Nursing, Faculty of Health Sciences, Medical University of Gdansk, Gdansk, Poland; 4 Neurology Department, St Adalbert Hospital, Copernicus PL Ltd., Gdansk, Poland; 5 Division of Clinical Trials and Biostatistics, Mayo Clinic, Jacksonville, FL, United States; 6 Department of Medicine, Division of Endocrinology, Diabetes and Metabolism, Mayo Clinic, Jacksonville, FL, United States

**Keywords:** antidiabetic medication, DDP4 inhibitors, Lewy body dementia, Parkinson's disease, SGLT2 inhibitors

## Abstract

**Introduction:**

Parkinson's disease (PD) and Dementia with Lewy Bodies (DLB) are progressive neurodegenerative disorders characterized by alpha-synuclein pathology. As current therapies largely target symptoms, there is a clear need for disease-modifying treatments. Emerging evidence links these disorders to glucose metabolism dysregulation, suggesting antidiabetic agents may hold therapeutic potential.

**Methods:**

This was a prospective, randomized, double-blind pilot study involving 12 subjects with PD or DLB to evaluate the tolerability and preliminary effects of a dipeptidyl peptidase 4 (DPP4) inhibitor (sitagliptin 100 mg) vs. sodium glucose like transporter 2 (SGLT2) inhibitor (dapagliflozin 10 mg) over a 4-week period, compared to a placebo. We assessed changes in Movement Disorder Society-Unified Parkinson's Disease Rating Scale (MDS-UPDRS) scores, cognitive function, and autonomic biomarkers. Both sitagliptin and dapagliflozin were well-tolerated, with no adverse events. While no significant changes were observed in cognitive scores, a significant difference was found in the change from baseline for MDS-UPDRS part 1 (*P* = 0.008), part 2 (*P* = 0.020), and total scores (*P* = 0.047) when comparing the three groups.

**Discussion:**

The dapagliflozin group showed more favorable trends in MDS-UPDRS scores. Limitations include small study size and duration as well as all participants had PD except one subject in the DPP4 inhibitor group with DLB. This pilot study suggests that these inhibitors are safe in PD and DLB patients. The preliminary findings, particularly the trends in the dapagliflozin group, provide a basis for larger-scale, long-term studies to confirm these potential symptomatic benefits and explore disease-modifying effects.

**Clinical trial registration:**

https://clinicaltrials.gov/study/NCT06263673, identifier: NCT06263673.

## 1 Introduction

Parkinson's disease (PD) and Dementia with Lewy Bodies (DLB) are progressive neurodegenerative disorders. PD and DLB are characterized by pathological accumulation of misfolded alpha-synuclein that eventually leads to motor symptoms such as bradykinesia, rigidity, tremor and postural instability as well as a broad spectrum of non-motor symptoms (1, 2). These non-motor manifestations—which include cognitive impairment, sleep disturbances, autonomic dysfunction, mood disorders, and hallucinations—can often precede motor signs and significantly impact patients' quality of life (3). Although both disorders share overlapping clinical and pathological features, DLB is typically associated with more pronounced and earlier cognitive decline and fluctuating attention, as well as visual hallucinations (4). The underlying mechanisms driving disease progression remain incompletely understood, and current treatment strategies are largely symptomatic, underscoring the urgent need for disease-modifying therapies (5). This unmet need has prompted increasing interest in novel treatment strategies targeting the underlying pathophysiological mechanisms of PD.

One promising area of research lies in the growing recognition of a link between PD and dysregulation of glucose metabolism (6, 7). A body of emerging evidence supports an interdependent relationship between neurodegeneration in PD and abnormalities in glucose homeostasis, with shared mechanisms including insulin resistance, impaired insulin signaling, neuroinflammation, mitochondrial dysfunction, and the accumulation of misfolded proteins (8–10). These overlapping features suggest that antidiabetic medications may hold therapeutic potential beyond glycemic control—possibly influencing neurodegenerative processes directly (10, 11). There are reports regarding the potential effects of other antidiabetic drugs in PD, but they require verification.

Among these agents, glucagon-like peptide-1 (GLP-1) receptor agonists have shown the most robust clinical evidence to date (12–14). Early trials in PD patients have demonstrated short-term improvements in motor—and in some cases, non-motor—symptoms. However, whether these improvements represent true disease-modifying effects or symptomatic relief remains unclear. Larger, longer-term studies are required to confirm their efficacy and define their role in altering disease progression.

Other classes of antidiabetic agents also show potential and warrant further investigation. Dipeptidyl peptidase-4 (DPP4) inhibitors, which decrease activity of the enzyme that breaks down active endogenous GLP-1, are well-tolerated and have shown neuroprotective effects in preclinical models (6, 15–17). In PD animal models, DPP4 inhibitors have been associated with reduced neuronal apoptosis and improved motor outcomes, while retrospective studies in humans suggest a possible reduced incidence of PD (8, 18, 19). GLP-1 and DPP4 are both present and active in the central nervous system (20). A non-human primate study demonstrated that oral administration of the DPP4 inhibitor, sitagliptin, significantly elevated both plasma and cerebrospinal fluid levels of GLP-1 (21).

Sodium-glucose cotransporter-2 (SGLT2) inhibitors represent another promising class. Originally developed to lower blood glucose by reducing renal glucose reabsorption, SGLT2 inhibitors also exert systemic benefits, including reduction of oxidative stress, inflammation, and cell death. (22–24). They are FDA-approved for cardiovascular and renal protection even in non-diabetic populations. Importantly, SGLT2 receptors are expressed throughout the central nervous system, and preclinical studies suggest these agents may exert neuroprotective effects in PD models by improving mitochondrial function, reducing oxidative damage, and preserving neuronal viability (25). Retrospective clinical data further suggest a potential benefit in lowering dementia risk. Mechanistically, SGLT2 inhibitors may support cognition through acetylcholinesterase inhibition, increased brain-derived neurotrophic factor (BDNF), and elevated ketone levels—each of which may contribute to neuroplasticity and neuronal resilience (22, 24).

Given this background, the aim of this pilot study was to test the hypothesis that prospective intervention with a DPP4 inhibitor or an SGLT2 inhibitor is well tolerated and may improve biomarkers relevant to Parkinson's disease DLB, in comparison to placebo. This study seeks to explore the therapeutic potential of these agents in modifying the disease process and to inform future trials targeting neurodegeneration through metabolic pathways.

## 2 Material and methods

### 2.1 Study subjects

A total of 11 PD patients and one DLB patient with prediabetes (or very mild early diabetes) who were seen at the Mayo Clinic in Jacksonville, Florida between May 14, 2024 and December 10, 2024 were included in this prospective, randomized, double-blind, pilot study. Patients were randomly assigned to one of three different treatment groups (sitagliptin 100 mg, dapagliflozin 10 mg, or placebo) in a 1:1:1 fashion, with a total of four patients in each group. Eligible participants were adults aged 45 years and older with a neurologist-confirmed diagnosis of PD or LBD at Mayo Clinic and stable neurological treatment for approximately 3 months. Patients were required to maintain a stable dopaminergic treatment regimen throughout the study period. Individuals must also have prediabetes or mild diabetes, defined using American Diabetes Association criteria as fasting glucose between 100 and 125 mg/dl or hemoglobin A1C between 5.7 and 6.4% for pre-diabetes, and values above these thresholds for diabetes. HbA1c was not reassessed at follow-up, as it reflects average glycemia over an approximately 3-month period and is unexpected to show meaningful changes within the study timeframe.

Given that 50%−80% of people with Parkinson's disease exhibit abnormal glucose tolerance, this requirement was not expected to limit recruitment. Exclusion criteria include the use of insulin or anti-diabetic medications other than metformin; contraindications to DPP4 or SGLT2 inhibitors such as allergy, prior angioedema, pancreatitis, active gallbladder disease, or renal impairment with an eGFR below 45; bleeding disorders, anticoagulant use, thrombocytopenia, or severe anemia; use of high-dose steroids; current systemic chemotherapy; pregnancy or breastfeeding; recent or recurrent urinary tract or yeast infections; and any additional condition that would pose safety concerns or compromise the interpretability of study data.

Information was collected regarding baseline patient characteristics (age, sex, race, ethnicity, diagnosis [PD or DLB], medical history [pre-diabetes or diabetes mellitus], medications. Neurological evaluation included MDS-UPDRS score (26) for part 1, part 2, part 3, and part 4, Mini Mental Status Examination (MMSE) score (27) done at baseline and approximately 4 weeks following baseline. Systolic blood pressure (SBP), diastolic blood pressure (DBP), and heart rate (HR) were measured at baseline and follow-up at (0 min) supine and after standing for 3, 6, 9, 12, and 15 min. Baseline and mean values for 0-15min were compared to follow up during treatment.

**Figure 1:**
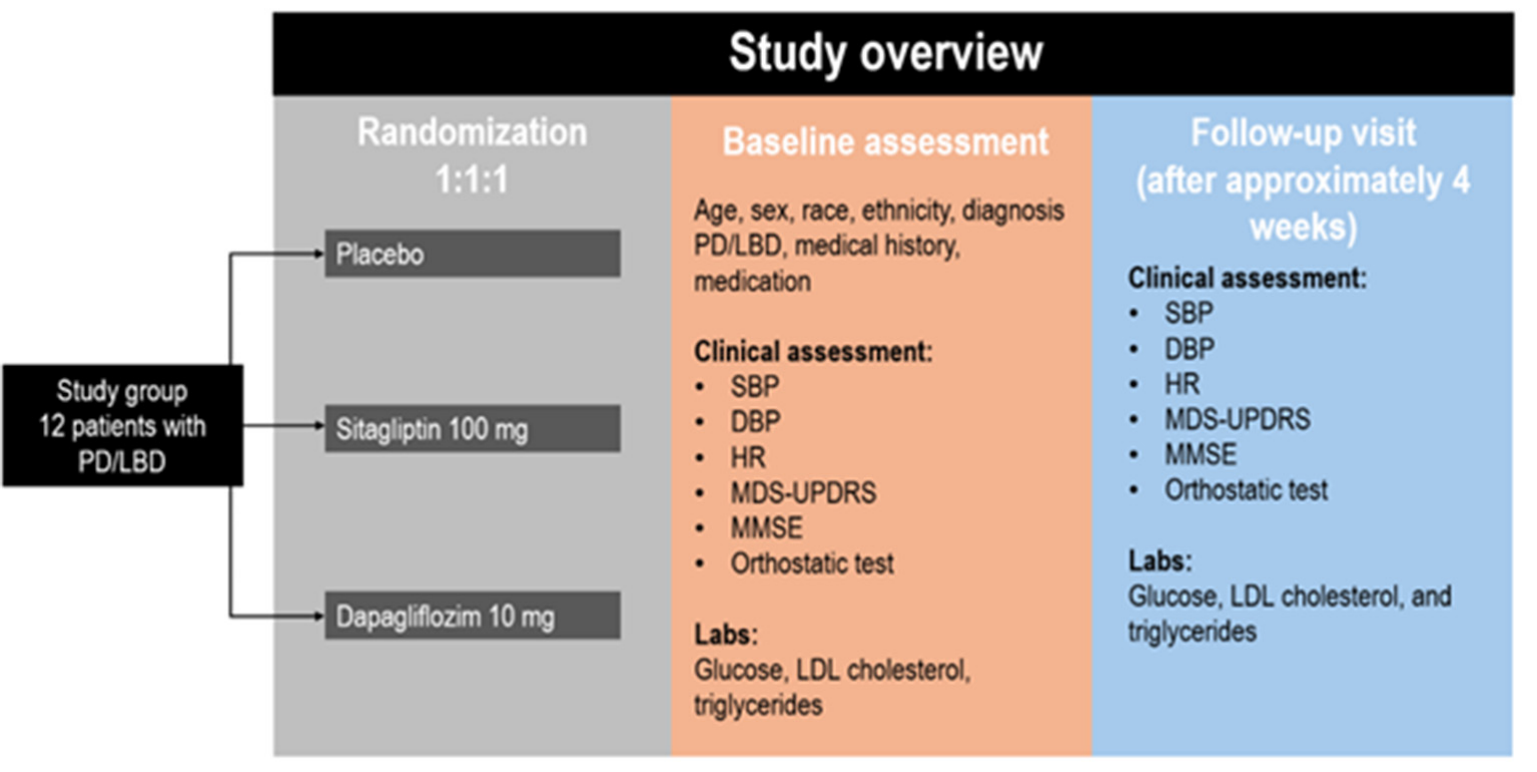
Outline of study protocol. PD, Parkinson's disease; LBD, Lewy body dementia; SBP, systolic blood pressure; DBP, diastolic blood pressure; HR, heart rate; MDS-UPDRS, Movement Disorder Society Unified Parkinson's Disease Rating Scale; MMSE, mini mental state examination.

The laboratory tests performed at baseline according to the study protocol included: glucose concentration, lipid profile, complete blood count, electrolyte panel, serum albumin, transaminase activity, and bilirubin levels. Glucose, LDL cholesterol and triglycerides, were then checked at follow-up visit. The schematic overview of the study protocol is illustrated in Figure 1.

### 2.2 Statistical analysis

All analyses were performed on the basis of the intention-to-treat principle. Continuous variables were summarized with median range and standard deviation. Categorical variables were summarized with number and percentage. Comparisons of baseline characteristics between the three groups were made using a one-way ANOVA (continuous variables) and Fisher's exact test (categorical variables). Comparisons of changes in outcomes from the baseline to follow-up visits (i.e., follow-up value minus baseline value) between the three groups were made using one-way ANOVA. Separately for each of the three treatment groups, comparisons of outcomes between the baseline and follow-up visits were made using a paired *t*-test. *P*-values <0.05 were considered as statistically significant. All statistical tests were two-sided. Statistical analyses were performed using SAS (version 9.4; SAS Institute, Inc., Cary, North Carolina).

## 3 Results

### 3.1 Group characteristics

The study included 12 patients. The median age was 66.3 years range (61.7–75.4) in the placebo group, 73.9 years (61.9–81.0) in the sitagliptin group, and 69.5 years (62.3–76.9) in the dapagliflozin group. Seven participants were male (58.3%). Ten patients had a medical history of prediabetes, and two had a diagnosis of mild early diabetes mellitus; three patients were treated with metformin. All patients were treated with levodopa, five received amantadine, and four were treated with antihypertensive medications. Baseline characteristics stratified by treatment group are presented in Table 1.

**Table 1:** Comparison of baseline patient characteristics between treatment groups.

Variable	Placebo group (*N* = 4)	Sitagliptin group (*N* = 4)	Dapagliflozin group (*N* = 4)	*P*-value
Age (years)	66.3 (61.7, 75.4)	73.9 (61.9, 81.0)	69.5 (62.3, 76.9)	0.58
Sex (male)	2 (50.0%)	2 (50.0%)	3 (75.0%)	1.00
Race (white)	4 (100.0%)	4 (100.0%)	3 (75.0%)	1.00
Ethnicity (not hispanic or latino)	4 (100.0%)	4 (100.0%)	4 (100.0%)	1.00
Diagnosis	
Parkinson's disease	4 (100.0%)	3 (75.0%)	4 (100.0%)	1.00
Lewy body dementia	0 (0.0%)	1 (25.0%)	0 (0.0%)	
Medical history	
Prediabetes	3 (75.0%)	3 (75.0%)	4 (100.0%)	1.00
Diabetes mellitus	1 (25.0%)	1 (25.0%)	0 (0.0%)	
Medications	
Metformin	1 (25.0%)	2 (50.0%)	0 (0.0%)	0.71
Levodopa	4 (100.0%)	4 (100.0%)	4 (100.0%)	1.00
Amantadine	1 (25.0%)	1 (25.0%)	3 (75.0%)	0.45
Anticholinergics	0 (0.0%)	0 (0.0%)	0 (0.0%)	1.00
Pimavanserin	0 (0.0%)	0 (0.0%)	0 (0.0%)	1.00
COMT	0 (0.0%)	0 (0.0%)	0 (0.0%)	1.00
Antihypertensive	2 (50.0%)	2 (50.0%)	0 (0.0%)	0.42
None	0 (0.0%)	0 (0.0%)	0 (0.0%)	1.00
Lab values	
Leukocytes	8.2 (6.5, 8.2)	6.5 (5.5, 7.2)	5.7 (3.7, 8.5)	0.21
Erythrocytes	5.1 (4.5, 5.5)	5.1 (4.1, 5.5)	4.5 (4.5, 5.0)	0.44
Hemoglobin	14.5 (13.3, 16.2)	16.1 (12.0, 17.4)	14.6 (14.0, 15.3)	0.74
Hematocrit	43.0 (41.0, 47.0)	47.0 (38.0, 49.0)	43.5 (40.0, 46.0)	0.71
MCV	90.0 (77.3, 93.0)	91.7 (89.8, 92.5)	93.9 (90.8, 97.6)	0.19
MCH	30.3 (25.1, 31.4)	30.9 (29.0, 32.5)	31.6 (30.7, 32.6)	0.28
MCHC	33.5 (32.5, 34.0)	34.1 (31.5, 35.2)	33.6 (32.7, 34.4)	0.91
RDW CV	13.4 (12.9, 14.6)	12.8 (12.2, 13.8)	12.2 (11.4, 13.9)	0.21
RDW SD	43.4 (40.5, 45.9)	43.2 (40.1, 47.2)	44.9 (39.1, 48.1)	0.95
Platelet count	217.5 (201.0, 314.0)	202.0 (165.0, 235.0)	219.5 (185.0, 294.0)	0.50
Mean platelet volume	10.7 (9.9, 11.4)	10.3 (9.7, 12.9)	10.5 (9.2, 10.8)	0.73
Neutrophils	64.5 (4.6, 74.5)	64.6 (54.0, 69.4)	59.2 (53.3, 2195.0)	0.41
Immature granulocytes	0.3 (0.2, 0.5)	0.5 (0.1, 0.9)	0.4 (0.3, 0.5)	0.66
Lymphocytes	18.2 (0.4, 31.5)	24.9 (21.6, 35.6)	31.7 (26.1, 42.1)	0.097
Monocytes	7.3 (6.3, 9.2)	7.0 (7.0, 8.6)	9.0 (6.9, 456.0)	0.40
Eosinophils	1.6 (0.2, 2.1)	1.7 (0.7, 2.7)	3.8 (2.8, 52.0)	0.31
Basophils	0.9 (0.5, 1.0)	0.6 (0.4, 0.8)	1.0 (0.0, 19.0)	0.41
Hemoglobin A1C	6.2 (5.8, 6.8)	6.7 (5.7, 7.2)	5.7 (4.7, 6.2)	0.11
Estimated average glucose	130.0 (120.0, 148.0)	145.5 (117.0, 160.0)	115.5 (78.0, 131.0)	0.11
Potassium	4.5 (3.7, 4.6)	4.2 (4.2, 4.5)	4.2 (3.7, 4.6)	0.80
Sodium	139.5 (136.0, 142.0)	139.5 (139.0, 142.0)	138.5 (138.0, 143.0)	0.88
Chloride	104.0 (101.0, 107.0)	103.0 (102.0, 105.0)	102.0 (101.0, 103.0)	0.32
Bicarbonate	25.0 (24.0, 25.0)	25.0 (21.0, 29.0)	24.0 (24.0, 27.0)	0.98
Anion gap	10.5 (8.0, 13.0)	12.0 (10.0, 13.0)	11.0 (11.0, 11.0)	0.58
BUN	14.0 (11.0, 22.0)	22.0 (12.0, 25.0)	18.0 (14.0, 21.0)	0.35
Creatinine	0.9 (0.9, 1.0)	0.8 (0.5, 1.0)	0.9 (0.9, 1.1)	0.31
Estimated GFR	82.5 (73.0, 90.0)	87.5 (63.0, 90.0)	82.0 (65.0, 90.0)	0.95
Total calcium	9.2 (8.5, 9.7)	9.5 (9.4, 9.8)	9.3 (8.9, 9.6)	0.34
Glucose	112.5 (95.0, 158.0)	139.5 (97.0, 215.0)	104.0 (78.0, 131.0)	0.30
Total protein	6.9 (6.4, 7.6)	7.1 (6.8, 7.6)	7.3 (7.2, 7.4)	0.44
Albumin	4.5 (3.5, 4.7)	4.5 (3.8, 4.9)	4.4 (4.3, 4.6)	0.91
Aspartate aminotransferase	22.5 (19.0, 24.0)	17.0 (14.0, 32.0)	30.0 (20.0, 52.0)	0.16
Alkaline phosphatase	88.0 (79.0, 94.0)	71.5 (67.0, 89.0)	60.0 (60.0, 80.0)	0.048
Alanine transaminase	19.5 (10.0, 38.0)	14.5 (8.0, 17.0)	19.5 (11.0, 31.0)	0.48
Total bilirubin	0.4 (0.2, 0.9)	0.6 (0.4, 1.5)	0.6 (0.5, 0.8)	0.49
Triglycerides	131.0 (102.0, 181.0)	113.0 (76.0, 315.0)	95.5 (53.0, 125.0)	0.45
Total cholesterol	153.0 (123.0, 233.0)	138.5 (87.0, 216.0)	145.5 (117.0, 190.0)	0.81
LDL cholesterol	83.5 (70.0, 115.0)	69.5 (30.0, 125.0)	77.5 (60.0, 128.0)	0.79
HDL cholesterol	46.0 (34.0, 87.0)	37.0 (28.0, 78.0)	46.5 (39.0, 52.0)	0.81
Non-HDL cholesterol	107.0 (89.0, 146.0)	106.5 (49.0, 138.0)	93.5 (76.0, 151.0)	0.88
Fasting (8 h or more)	2 (50.0%)	2 (50.0%)	4 (100.0%)	0.42

### 3.2 Neurological outcomes

**Figure 2:**
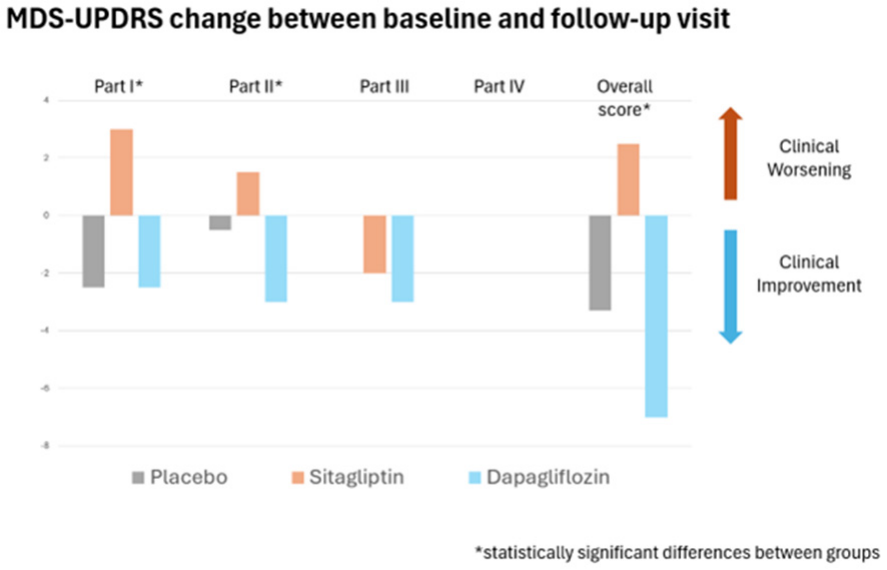
The change in the MDS-UPDRS score from baseline to follow-up was compared between the study groups. MDS-UPDRS, Movement Disorder Society Unified Parkinson's Disease Rating Scale.

In the sitagliptin group, there was a significant increase in MDS-UPDRS part 1 score from baseline to follow-up (Median change: 3.0, *P* = 0.035). In the placebo group, though not quite statistically significant, there was a decrease in MDS-UPDRS part 1 score from baseline to follow-up (Median change: −2.5, *P* = 0.063). Additionally, though not quite statistically significant, in the dapagliflozin group there was a decrease in MDS-UPDRS part 2 score from baseline to follow-up (Median change: −3.0, *P* = 0.063). When comparing changes in these outcomes from baseline to follow-up between placebo, sitagliptin and dapagliflozin groups, there was a significant difference regarding MDS-UPDRS part 1 score (Median change: −2.5 vs. 3.0 vs. −2.5, *P* = 0.008) and MDS-UPDRS part 2 score (Median: −0.5 vs. 1.5 vs. 3.0, *P* = 0.020) and total MDS-UPDRS (Median: −3.5 vs. 2.5 vs. −7.0, *P* = 0.047). Change in MDS-UPDRS is visualized in Figure 2. Comparisons of changes in MDS-UPDRS score and MMSE score from baseline to follow-up between the three groups, as well as an assessment of changes in these outcomes from baseline to follow-up within each group, are displayed in Table 2.

**Table 2:** Comparisons of changes in MDS-UPDRS and MMSE from baseline to follow-up within and between treatment groups.

Outcome	Placebo group (*N* = 4)	Sitagliptin group (*N* = 4)	Dapagliflozin group (*N* = 4)	*P*-value
MDS-UPDRS score – part 1	
Baseline	8.0 (2.0, 22.0)	7.5 (2.0, 16.0)	7.0 (6.0, 9.0)	0.008
Follow-up	5.5 (1.0, 18.0)	10.0 (4.0, 20.0)	4.0 (2.0, 7.0)	
Follow-up minus baseline	−2.5 (−4.0, −1.0)	3.0 (1.0, 4.0)	−2.5 (−7.0, 0.0)	
*P*-value: follow-up vs. baseline	0.063	0.035	0.13	
MDS-UPDRS score – part 2	
Baseline	6.5 (1.0, 23.0)	10.0 (4.0, 17.0)	9.0 (4.0, 19.0)	0.020
Follow-up	7.0 (0.0, 20.0)	12.5 (4.0, 19.0)	6.5 (3.0, 15.0)	
Follow-up minus baseline	−0.5 (−3.0, 1.0)	1.5 (0.0, 4.0)	−3.0 (−4.0, 0.0)	
*P*-value: follow-up vs. baseline	0.44	0.13	0.063	
MDS-UPDRS score – part 3	
Baseline	25.5 (18.0, 39.0)	25.5 (19.0, 37.0)	38.0 (24.0, 50.0)	0.19
Follow-up	25.5 (18.0, 41.0)	21.0 (18.0, 37.0)	36.5 (10.0, 47.0)	
Follow-up minus baseline	0.0 (0.0, 2.0)	−2.0 (−6.0, 0.0)	−3.0 (−14.0, 0.0)	
*P*-value: follow-up vs. baseline	0.39	0.16	0.20	
MDS-UPDRS score – part 4	
Baseline	0.5 (0.0, 2.0)	0.0 (0.0, 0.0)	0.0 (0.0, 4.0)	0.33
Follow-up	0.0 (0.0, 1.0)	0.0 (0.0, 8.0)	0.0 (0.0, 1.0)	
Follow-up minus Baseline	0.0 (−2.0, 0.0)	0.0 (0.0, 8.0)	0.0 (−4.0, 1.0)	
*P*-value: follow-up vs. baseline	0.39	0.39	0.55	
MDS-UPDRS total score	
Baseline	49.5 (22.0, 67.0)	47.5 (32.0, 54.0)	52.5 (43.0, 76.0)	0.047
Follow-up	47.5 (20.0, 60.0)	51.0 (33.0, 62.0)	48.5 (15.0, 67.0)	
Follow-up minus baseline	−3.5 (−7.0, 1.0)	2.5 (1.0, 10.0)	−7.0 (−28.0, −3.0)	
*P*-value: follow-up vs. baseline	0.16	0.14	0.14	
MMSE score	
Baseline	26.5 (23.0, 29.0)	26.0 (23.0, 28.0)	28.0 (25.0, 30.0)	0.67
Follow-up	28.0 (24.0, 30.0)	28.0 (24.0, 30.0)	28.0 (28.0, 29.0)	
Follow-up minus baseline	1.5 (−1.0, 3.0)	1.5 (0.0, 4.0)	0.5 (−2.0, 3.0)	
*P*-value: follow-up vs. baseline	0.24	0.13	0.70	

### 3.3 Orthostatic blood pressure

Comparisons among the three groups demonstrated a significant difference in the change in orthostatic heart rate (HR) at 15 min relative to 0 min between baseline and follow-up visits (median change: 8.0 in the placebo group, 2.0 in the sitagliptin group, and −4.5 in the dapagliflozin group; *P* = 0.030). In addition, although not statistically significant, the change in orthostatic systolic blood pressure (SBP) at 15 min relative to 0 min differed between groups (median change: −9.5, 2.0, and 15.0, respectively; *P* = 0.075).

No other significant between-group differences in changes from baseline to follow-up were observed for the assessed outcome measures (all *P* ≥ 0.092; Table 3). Comparisons of baseline-to-follow-up changes in orthostatic SBP, diastolic blood pressure (DBP), HR, mean arterial pressure (MAP), and supine MAP within and between treatment groups are summarized in Table 3 with no statistically significant differences detected among the three treatment groups (all *P* ≥ 0.061). Detailed SBP, DBP, and HR data stratified by treatment group are provided in Table 3.

**Table 3:** Detailed SBP, DBP, and HR data stratified by treatment group.

Outcome	Placebo group (*N* = 4)	Sitagliptin group (*N* = 4)	Dapagliflozin group (*N* = 4)	*P*-value
Orthostatic systolic blood pressure (SBP)				
**0 min**				
Baseline	126.0 (100.0, 145.0)	138.5 (128.0, 159.0)	141.0 (121.0, 161.0)	0.56
Follow-up	124.5 (106.0, 143.0)	123.5 (109.0, 145.0)	131.5 (124.0, 155.0)	
Follow-up minus baseline	−6.5 (−29.0, 43.0)	−11.0 (−38.0, −3.0)	−5.0 (−18.0, 6.0)	
*P*-value: follow-up vs. baseline	0.99	0.14	0.35	
**15 min**				
Baseline	133.5 (117.0, 146.0)	132.5 (125.0, 153.0)	122.0 (117.0, 152.0)	0.061
Follow-up	119.5 (116.0, 121.0)	125.0 (105.0, 139.0)	134.0 (124.0, 155.0)	
Follow-up minus baseline	−14.0 (−25.0, −1.0)	−10.0 (−34.0, 5.0)	5.0 (3.0, 21.0)	
*P*-value: follow-up vs. baseline	0.098	0.17	0.14	
**Mean of 3–15 min minus 0**				
Baseline	128.0 (112.2, 137.7)	137.4 (128.7, 157.2)	129.0 (120.5, 162.0)	0.38
Follow-up	121.0 (118.5, 127.7)	127.1 (108.3, 142.7)	128.9 (121.2, 155.0)	
Follow-up minus baseline	−2.5 (−19.0, 6.3)	−9.6 (−37.7, 1.3)	−1.1 (−7.0, 2.7)	
15.6-7.8,-1.5498.8pt*P*-value: follow-up vs. baseline	0.51	0.23	0.50	
Orthostatic diastolic blood pressure (DBP)				
**0 min**				
Baseline	79.5 (69.0, 88.0)	83.0 (74.0, 91.0)	84.5 (80.0, 93.0)	0.47
Follow-up	76.5 (73.0, 81.0)	78.0 (69.0, 90.0)	85.0 (81.0, 102.0)	
Follow-up minus baseline	−3.5 (−14.0, 12.0)	−6.5 (−9.0, 6.0)	1.5 (−1.0, 9.0)	
*P*-value: follow-up vs. baseline	0.70	0.20	0.30	
**15 min**				
Baseline	73.5 (71.0, 88.0)	85.5 (66.0, 98.0)	80.5 (78.0, 104.0)	0.079
Follow-up	80.5 (75.0, 89.0)	77.0 (66.0, 85.0)	88.0 (82.0, 96.0)	
Follow-up minus baseline	5.0 (1.0, 8.0)	−7.5 (−15.0, 0.0)	3.5 (−8.0, 12.0)	
*P*-value: follow-up vs. baseline	0.050	0.18	0.55	
**Mean of 3–15 min minus 0**				
Baseline	77.5 (72.8, 90.7)	85.0 (74.7, 94.2)	82.2 (80.8, 99.2)	0.33
Follow-up	79.6 (68.8, 83.8)	80.4 (69.8, 85.2)	84.4 (81.5, 96.5)	
Follow-up minus baseline	−3.5 (−6.8, 7.2)	−4.8 (−14.3, 1.0)	0.8 (−2.7, 3.7)	
15.6-7.8,-1.5498.8pt*P*-value: follow-up vs. baseline	0.63	0.21	0.69	
Orthostatic heart rate (HR)				
**0 min**				
Baseline	87.0 (68.0, 107.0)	70.5 (65.0, 80.0)	67.0 (54.0, 98.0)	0.36
Follow-up	77.0 (70.0, 94.0)	67.5 (60.0, 78.0)	69.5 (62.0, 91.0)	
Follow-up minus baseline	−8.0 (−17.0, 2.0)	−3.5 (−14.0, 8.0)	2.0 (−7.0, 9.0)	
*P*-value: follow-up vs. baseline	0.18	0.56	0.74	
**15 min**				
Baseline	88.0 (75.0, 103.0)	76.5 (64.0, 87.0)	69.0 (59.0, 100.0)	0.86
Follow-up	87.5 (77.0, 103.0)	72.0 (62.0, 93.0)	69.5 (58.0, 87.0)	
Follow-up minus baseline	−1.5 (−9.0, 13.0)	1.5 (−14.0, 6.0)	−3.0 (−13.0, 6.0)	
P-value: follow-up vs. baseline	0.96	0.80	0.47	
**Mean of 3–15 min minus 0**				
Baseline	88.8 (68.5, 103.2)	75.3 (65.3, 84.8)	71.0 (57.0, 99.3)	0.93
Follow-up	85.5 (76.3, 94.8)	69.5 (60.7, 85.7)	69.8 (59.8, 90.2)	
Follow-up minus baseline	−2.3 (−10.3, 7.8)	−1.5 (−13.2, 0.8)	−3.6 (−9.2, 7.7)	
15.6-7.8,-1.5498.8pt*P*-value: follow-up vs. baseline	0.74	0.33	0.58	
Orthostatic MAP				
**0 min**				
Baseline	96.7 (79.3, 103.7)	98.8 (97.3, 113.7)	103.3 (93.7, 115.7)	0.59
Follow-up	92.2 (84.7, 101.7)	95.5 (82.3, 103.7)	100.0 (96.3, 119.7)	
Follow-up minus baseline	−4.5 (−19.0, 22.3)	−9.3 (−16.0, 3.0)	1.3 (−6.7, 4.0)	
*P*-value: follow-up vs. baseline	0.88	0.14	1.00	
**Mean of 3–15 min minus 0**				
Baseline	93.8 (86.9, 106.3)	102.4 (92.7, 115.2)	97.3 (94.9, 120.1)	0.32
Follow-up	94.9 (85.4, 95.4)	96.0 (82.7, 104.3)	99.3 (94.7, 116.0)	
Follow-up minus baseline	−2.8 (−10.9, 6.2)	−6.4 (−22.1, 1.1)	0.1 (−4.1, 3.3)	
*P*-value: follow-up vs. baseline	0.52	0.20	0.94	

### 3.4 Tolerance and adverse effects

There were no adverse effects reported during the course of the study. All three interventions were well tolerated.

### 3.5 Analysis excluding one DLB patient

An analysis excluding the patient diagnosed with DLB was also performed; this did not significantly change any of the aforementioned results. Detailed outcomes of this analysis are available in the Supplementary Materials (Supplementary Tables 3–4)

## 4 Discussion

This pilot study assessed the tolerability and potential clinical benefits of DPP4 and SGLT2 inhibitors in patients with PD or DLB. While GLP-1 receptor agonists have garnered increasing attention in the context of PD, DPP4 and SGLT2 inhibitors remain less extensively studied, despite encouraging preclinical data suggesting possible neuroprotective effects (7, 8, 28).

In this study, both drug classes were well tolerated, with no adverse events reported. Biochemical analyses revealed no significant abnormalities, supporting a favorable safety profile. These results suggest that larger-scale studies involving PD patients treated with DPP4 and SGLT2 inhibitors could be safely undertaken.

Nonetheless, long-term treatment with agents such as GLP-1 receptor agonists and SGLT2 inhibitors may present challenges in PD populations. Notably, significant weight loss—common in patients with advanced PD—can be exacerbated by these drugs. Weight loss is not only associated with disease progression (29) but also can be complication from levodopa therapy especially continues infusions such as levodopa-carbidopa intestinal gel (LCIG) (5). Weight loss may be intensified by these pharmacologic agents (GLP-1 agonist which are FDA approved for weight loss and less so SGLT2 inhibitors which are not FDA approved for weight loss) (30, 31). Conversely, deep brain stimulation (DBS) is often associated with weight gain (32). Therefore, when enrolling patients into trials involving these drugs, it is essential to exercise strict caution and closely monitor changes in body weight.

It is well-established in the literature that SGLT2 inhibitors promote mild to moderate weight loss primarily through continuous urinary caloric excretion (glycosuria) and mild fluid shifts (33, 34). Conversely, DPP4 inhibitors are widely recognized as weight-neutral (35). The beneficial effects observed in the DPP4 inhibitor cohort in our study therefore strongly suggest a direct, weight-independent mechanism of action—likely mediated through the stabilization of incretin hormones (GLP-1 and GIP) and subsequent modulation of systemic and localized inflammatory pathways (35, 36).

While the secondary benefits of weight loss cannot be entirely ruled out for the SGLT2 inhibitor group, accumulating evidence points toward pleiotropic, direct effects for this class as well. For instance, SGLT2 inhibition shifts systemic metabolism toward ketone body utilization (β-hydroxybutyrate), which serves as an efficient, neuroprotective energy substrate independent of body mass changes (28, 37). Furthermore, both drug classes have demonstrated direct anti-inflammatory and endothelial-stabilizing properties in vitro, (38, 39). Future studies incorporating rigorous, longitudinal body weight tracking are warranted to definitively isolate the direct effects of SGLT2 and DPP4 inhibitors from the indirect benefits of weight loss.

Despite the small sample size and limited duration of our observation, some differences emerged between treatment groups. Patients receiving SGLT2 inhibitors showed more favorable changes in MDS-UPDRS scores, particularly in domains related to motor function and activities of daily living (both motor and non-motor). In contrast, no definitive clinical benefit was observed in the DPP4 inhibitor group. These findings must be interpreted cautiously, especially given the inclusion of a patient with DLB in the DPP4 group—a condition associated with different clinical phenotype (40). Although in our group the results were maintained even after excluding the patient with DLB, it should be taken into account that this may have skewed the results and underscored the importance of using more balanced study populations in future research.

Our findings suggest a potentially beneficial effect of SGLT2 inhibitors on parkinsonian symptoms. Supporting this, Rozani et al. (18) reported that individuals with diabetes treated with GLP-1 receptor agonists, DPP4 inhibitors, SGLT2 inhibitors, or meglitinides had a significantly lower risk of developing PD compared to those using metformin—with the most substantial and statistically significant reduction observed in the SGLT2 inhibitor group. Similar findings were reported in a nationwide population-based study by Kim et al. (41).

Current evidence on SGLT2 inhibitors largely pertains to PD risk reduction rather than their impact on clinical symptoms or progression. Effects on motor function remain inconclusive in clinical trials. Our study contributes preliminary evidence suggesting symptomatic benefits. Similar to an analysis by Jaiswal et al. (31), we do not confirm changes in cognition

Another challenge is detecting true disease-modifying effects in PD which remains inherently difficult. The short observation period of this study further limits conclusions to potential symptomatic improvements only. Future investigations should incorporate disease-specific objective biomarkers—such as functional neuroimaging or alpha-synuclein quantification—to better evaluate disease-modifying potential (42, 43).

Emerging evidence indicates that blood brain barrier (BBB) dapagliflozin is highly lipophilic and successfully permeates the BBB, enabling direct central neuroprotective effects such as the attenuation of microglial activation, reactive oxygen species generation, and acetylcholinesterase levels (28, 44). Consequently, it is critical to evaluate whether the observed improvements in motor scores are primarily driven by these direct, centrally mediated mechanisms or by indirect, peripheral metabolic improvements—such as enhanced systemic glycemic control, reduced systemic inflammation, and the neuroprotective shift toward ketone body utilization. Similarly, while certain DPP4 inhibitors demonstrate BBB penetrance, their neuroprotective efficacy is largely attributed to the peripheral stabilization of incretins (GLP-1 and GIP) and interactions at the neurovascular unit, which subsequently modulate central inflammatory tone without requiring direct deep-brain penetration (45, 46). Delineating the proportional contributions of these central vs. peripheral pathways remains a vital avenue for future neuro-metabolic research.

Patient selection is another critical factor to consider. PD is a clinically heterogeneous disorder, encompassing a wide range of motor and non-motor symptoms. Prior research suggests that autonomic dysfunction, frequently observed in both PD and DLB (47, 48), may be predictive of glycemic dysregulation (19, 49–51). Assessing patients for autonomic disorders may be crucial during group selection, as their presence could represent a potential confounding factor. Another potential challenge is assessing the impact of dysautonomia severity on the effectiveness of potential interventions. In the present study, all participants were assessed for orthostatic hypotension. Although some variation in autonomic function was observed, none met the diagnostic criteria for orthostatic hypotension (52) limiting our ability to assess this variable. Future studies should examine how autonomic symptoms might influence the response to antidiabetic therapies, as differential effects may exist between patients with and without such dysfunction.

The primary limitation of this study is its small sample size. While appropriate for a pilot investigation, the limited number of participants reduces statistical power, increasing the risk of a type II error (i.e., a false-negative finding). As such, a lack of statistically significant findings should not be interpreted as evidence of no effect. Consequently, the robustness and reproducibility of the observed effects should be interpreted with caution. Larger, adequately powered studies are warranted to confirm these results and strengthen the external validity of the conclusions. In addition, this study is inherently too brief to evaluate definitive disease-modifying effects in a chronic, progressive neurodegenerative condition such as Parkinson's disease. Therefore, any clinical or biological signals observed during this period should be interpreted cautiously and regarded as preliminary. Determining whether this approach truly modifies the natural course of the disease will require future longitudinal studies with substantially longer follow-up periods. Additionally, future studies should include HbA1c measurements to account for the potential confounding effects of antidiabetic medications on glycemic control, which may subsequently influence motor outcomes. Another limitation of this study is the small sample size, which precluded meaningful sex- stratified analyses. As a result, potential sex-specific differences in disease progression and treatment response could not be adequately assessed. This may limit the generalizability of the findings, and future studies with larger and more balanced cohorts are needed to address this issue. An additional limitation of the present study is the absence of comprehensive body weight data across the experimental timeline. Dapagliflozin in prior clinical trials has been shown to be associated with approximately 1 kg or less of weight loss by week 4, however this amount can increase after a longer duration of therapy to 3 kg after 24 weeks (53). Distinguishing the direct pharmacological effects of these treatments from the secondary metabolic benefits of weight loss is crucial, particularly given the distinct phenotypic profiles of the drugs utilized. Despite these constraints, the study reinforces the safety of both DPP4 and SGLT2 inhibitors in PD and highlights several key considerations for the design of future, larger-scale clinical trials.

This pilot study explored the potential of DPP4 and SGLT2 inhibitors to affect biomarkers in PD and DLB. Despite a small sample size, the findings offer early insights into the tolerability and possible benefits of these diabetes drugs in PD and DLB. Notable trends, including changes in MDS-UPDRS scores and orthostatic heart rate, suggest a need for larger, long-term studies to confirm efficacy and assess potential disease-modifying effects.

## Data Availability

The raw data supporting the conclusions of this article will be made available by the authors, without undue reservation.
