# Supplementary material for: Anti-diabetic medications to fight Parkinson's disease and dementia with Lewy bodies: a pilot study
Source: Front Neurol. 2026 May 29;17:1825229. doi: 10.3389/fneur.2026.1825229 (PMC13274524; doi:10.3389/fneur.2026.1825229)
Supplement: Supplementary file 1 [file Supplementary_file_1.docx]

**Supplementary material**

**Anti-Diabetic Medications to Fight Parkinson’s Disease and Lewy Body Dementia a pilot study**

**Contents**

[**Supplementary Table 1:** Comparisons of orthostatic systolic blood pressure, orthostatic diastolic blood pressure, orthostatic heart rate, orthostatic MAP, and supine MAP from baseline to follow-up within and between treatment groups 2](#_Toc219290521)

[**Supplementary Table 2:** Comparison of baseline patient characteristics between treatment groups 8](#_Toc219290522)

[**Supplemental Table 3:** Comparison of baseline patient characteristics between treatment groups when excluding the 1 Lewy body dementia patient from Group 11](#_Toc219290523)

[**Supplemental Table 4:** Comparisons of changes in MDS-UPDRS and MMSE from baseline to follow-up within and between treatment groups when excluding the 1 Lewy body dementia patient from Group B 14](#_Toc219290524)

[**Supplemental Table 5:** Comparisons of changes in orthostatic systolic blood pressure, orthostatic diastolic blood pressure, orthostatic heart rate, orthostatic MAP, and supine MAP from baseline to follow-up within and between treatment groups when excluding the 1 Lewy body dementia patient from Group B 16](#_Toc219290525)

# **Supplementary Table 1:** Comparisons of orthostatic systolic blood pressure, orthostatic diastolic blood pressure, orthostatic heart rate, orthostatic MAP, and supine MAP from baseline to follow-up within and between treatment groups

|  | Placebo group (N=4) | | Sitagliptin group (N=4) | | Dapagliflozin group (N=4) | |  |
| --- | --- | --- | --- | --- | --- | --- | --- |
| Outcome | N | Median (minimum, maximum) or p-value | N | Median (minimum, maximum) or p-value | N | Median (minimum, maximum) or p-value | P-value for comparison between groups of change between time points |
| **Orthostatic systolic blood pressure (SBP)** |  |  |  |  |  |  |  |
| 0 minutes |  |  |  |  |  |  |  |
| Baseline | 4 | 126.0 (100.0, 145.0) | 4 | 138.5 (128.0, 159.0) | 4 | 141.0 (121.0, 161.0) |  |
| Follow-up | 4 | 124.5 (106.0, 143.0) | 4 | 123.5 (109.0, 145.0) | 4 | 131.5 (124.0, 155.0) |  |
| Follow-up minus Baseline | 4 | -6.5 (-29.0, 43.0) | 4 | -11.0 (-38.0, -3.0) | 4 | -5.0 (-18.0, 6.0) | 0.56 |
| P-value: Follow-up vs. Baseline |  | 0.99 |  | 0.14 |  | 0.35 |  |
| 3 minutes |  |  |  |  |  |  |  |
| Baseline | 4 | 124.0 (102.0, 139.0) | 4 | 148.0 (135.0, 155.0) | 4 | 128.5 (123.0, 181.0 |  |
| Follow-up | 4 | 123.0 (122.0, 133.0) | 4 | 138.0 (107.0, 145.0) | 4 | 128.5 (118.0, 166.0) |  |
| Follow-up minus Baseline | 4 | 3.0 (-15.0, 21.0) | 4 | -8.5 (-46.0, 5.0) | 4 | -3.5 (-15.0, 2.0) | 0.36 |
| P-value: Follow-up vs. Baseline |  | 0.74 |  | 0.28 |  | 0.26 |  |
| 6 minutes |  |  |  |  |  |  |  |
| Baseline | 4 | 133.0 (108.0, 138.0) | 4 | 139.0 (123.0, 156.0) | 4 | 128.0 (123.0, 177.0) |  |
| Follow-up | 4 | 120.5 (107.0, 133.0) | 4 | 127.0 (113.0, 138.0) | 4 | 129.0 (121.0, 163.0) |  |
| Follow-up minus Baseline | 4 | -7.5 (-15.0, -1.0) | 4 | -9.0 (-41.0, 7.0) | 4 | -2.5 (-14.0, 5.0) | 0.63 |
| P-value: Follow-up vs. Baseline |  | 0.084 |  | 0.31 |  | 0.46 |  |
| 9 minutes |  |  |  |  |  |  |  |
| Baseline | 4 | 124.5 (118.0, 137.0) | 4 | 142.5 (129.0, 158.0) | 4 | 124.5 (118.0, 155.0) |  |
| Follow-up | 4 | 116.5 (115.0, 123.0) | 4 | 127.5 (110.0, 144.0) | 4 | 131.0 (117.0, 144.0) |  |
| Follow-up minus Baseline | 4 | -8.0 (-14.0, -3.0) | 4 | -14.0 (-39.0, 4.0) | 4 | 1.5 (-11.0, 9.0) | 0.21 |
| P-value: Follow-up vs. Baseline |  | 0.059 |  | 0.17 |  | 0.96 |  |
| 12 minutes |  |  |  |  |  |  |  |
| Baseline | 4 | 128.0 (107.0, 140.0) | 4 | 133.5 (113.0, 162.0) | 4 | 130.0 (121.0, 146.0) |  |
| Follow-up | 4 | 128.5 (106.0, 139.0) | 4 | 121.5 (106.0, 145.0) | 4 | 122.5 (117.0, 147.0) |  |
| Follow-up minus Baseline | 4 | -6.5 (-20.0, 32.0) | 4 | -11.0 (-28.0, 2.0) | 4 | -5.5 (-8.0, 1.0) | 0.61 |
| P-value: Follow-up vs. Baseline |  | 0.99 |  | 0.17 |  | 0.11 |  |
| 15 minutes |  |  |  |  |  |  |  |
| Baseline | 4 | 133.5 (117.0, 146.0) | 4 | 132.5 (125.0, 153.0) | 4 | 122.0 (117.0, 152.0) |  |
| Follow-up | 4 | 119.5 (116.0, 121.0) | 4 | 125.0 (105.0, 139.0) | 4 | 134.0 (124.0, 155.0) |  |
| Follow-up minus Baseline | 4 | -14.0 (-25.0, -1.0) | 4 | -10.0 (-34.0, 5.0) | 4 | 5.0 (3.0, 21.0) | 0.061 |
| P-value: Follow-up vs. Baseline |  | 0.098 |  | 0.17 |  | 0.14 |  |
| Mean of 0 to 15 minutes |  |  |  |  |  |  |  |
| Baseline | 4 | 128.0 (112.2, 137.7) | 4 | 137.4 (128.7, 157.2 | 4 | 129.0 (120.5, 162.0) |  |
| Follow-up | 4 | 121.0 (118.5, 127.7) | 4 | 127.1 (108.3, 142.7) | 4 | 128.9 (121.2, 155.0) |  |
| Follow-up minus Baseline | 4 | -2.5 (-19.0, 6.3) | 4 | -9.6 (-37.7, 1.3) | 4 | -1.1 (-7.0, 2.7) | 0.38 |
| P-value: Follow-up vs. Baseline |  | 0.51 |  | 0.23 |  | 0.50 |  |
|  |  |  |  |  |  |  |  |
| **Orthostatic diastolic blood pressure (DBP)** |  |  |  |  |  |  |  |
| 0 minutes |  |  |  |  |  |  |  |
| Baseline | 4 | 79.5 (69.0, 88.0) | 4 | 83.0 (74.0, 91.0) | 4 | 84.5 (80.0, 93.0) |  |
| Follow-up | 4 | 76.5 (73.0, 81.0) | 4 | 78.0 (69.0, 90.0) | 4 | 85.0 (81.0, 102.0) |  |
| Follow-up minus Baseline | 4 | -3.5 (-14.0, 12.0) | 4 | -6.5 (-9.0, 6.0) | 4 | 1.5 (-1.0, 9.0) | 0.47 |
| P-value: Follow-up vs. Baseline |  | 0.70 |  | 0.20 |  | 0.30 |  |
| 3 minutes |  |  |  |  |  |  |  |
| Baseline | 4 | 77.5 (52.0, 93.0) | 4 | 91.5 (65.0, 97.0) | 4 | 83.0 (80.0, 106.0) |  |
| Follow-up | 4 | 83.0 (62.0, 86.0) | 4 | 85.5 (67.0, 88.0) | 4 | 84.5 (83.0, 98.0) |  |
| Follow-up minus Baseline | 4 | -2.5 (-10.0, 29.0) | 4 | -7.5 (-23.0, 19.0) | 4 | 1.0 (-8.0, 4.0) | 0.74 |
| P-value: Follow-up vs. Baseline |  | 0.72 |  | 0.33 |  | 0.87 |  |
| 6 minutes |  |  |  |  |  |  |  |
| Baseline | 4 | 79.0 (71.0, 88.0) | 4 | 83.0 (70.0, 92.0) | 4 | 83.0 (81.0, 108.0) |  |
| Follow-up | 4 | 74.5 (65.0, 81.0) | 4 | 85.0 (71.0, 87.0) | 4 | 84.5 (81.0, 105.0) |  |
| Follow-up minus Baseline | 4 | -7.0 (-12.0, 4.0) | 4 | -1.5 (-12.0, 15.0) | 4 | 0.0 (-4.0, 4.0) | 0.56 |
| P-value: Follow-up vs. Baseline |  | 0.20 |  | 0.62 |  | 1.00 |  |
| 9 minutes |  |  |  |  |  |  |  |
| Baseline | 4 | 82.5 (76.0, 95.0) | 4 | 87.5 (78.0, 92.0) | 4 | 82.0 (81.0, 92.0) |  |
| Follow-up | 4 | 78.0 (67.0, 85.0) | 4 | 79.0 (67.0, 87.0) | 4 | 84.0 (76.0, 90.0) |  |
| Follow-up minus Baseline | 4 | -8.5 (-10.0, -1.0) | 4 | -8.0 (-15.0, -2.0) | 4 | -0.5 (-6.0, 4.0) | 0.11 |
| P-value: Follow-up vs. Baseline |  | 0.042 |  | 1.00 |  | 0.75 |  |
| 12 minutes |  |  |  |  |  |  |  |
| Baseline | 4 | 84.5 (75.0, 92.0) | 4 | 85.5 (79.0, 99.0) | 4 | 82.5 (80.0, 92.0) |  |
| Follow-up | 4 | 84.0 (63.0, 91.0) | 4 | 78.5 (68.0, 84.0) | 4 | 83.0 (81.0, 88.0) |  |
| Follow-up minus Baseline | 4 | -3.5 (-23.0, 16.0) | 4 | -11.5 (-16.0, -1.0) | 4 | -0.5 (-4.0, 3.0) | 0.44 |
| P-value: Follow-up vs. Baseline |  | 0.69 |  | 0.067 |  | 0.75 |  |
| 15 minutes |  |  |  |  |  |  |  |
| Baseline | 4 | 73.5 (71.0, 88.0) | 4 | 85.5 (66.0, 98.0) | 4 | 80.5 (78.0, 104.0) |  |
| Follow-up | 4 | 80.5 (75.0, 89.0) | 4 | 77.0 (66.0, 85.0) | 4 | 88.0 (82.0, 96.0) |  |
| Follow-up minus Baseline | 4 | 5.0 (1.0, 8.0) | 4 | -7.5 (-15.0, 0.0) | 4 | 3.5 (-8.0, 12.0) | 0.079 |
| P-value: Follow-up vs. Baseline |  | 0.050 |  | 0.18 |  | 0.55 |  |
| Mean of 0 to 15 minutes |  |  |  |  |  |  |  |
| Baseline | 4 | 77.5 (72.8, 90.7) | 4 | 85.0 (74.7, 94.2) | 4 | 82.2 (80.8, 99.2) |  |
| Follow-up | 4 | 79.6 (68.8, 83.8) | 4 | 80.4 (69.8, 85.2) | 4 | 84.4 (81.5, 96.5) |  |
| Follow-up minus Baseline | 4 | -3.5 (-6.8, 7.2) | 4 | -4.8 (-14.3, 1.0) | 4 | 0.8 (-2.7, 3.7) | 0.33 |
| P-value: Follow-up vs. Baseline |  | 0.63 |  | 0.21 |  | 0.69 |  |
|  |  |  |  |  |  |  |  |
| **Orthostatic heart rate (HR)** |  |  |  |  |  |  |  |
| 0 minutes |  |  |  |  |  |  |  |
| Baseline | 4 | 87.0 (68.0, 107.0) | 4 | 70.5 (65.0, 80.0) | 4 | 67.0 (54.0, 98.0) |  |
| Follow-up | 4 | 77.0 (70.0, 94.0) | 4 | 67.5 (60.0, 78.0) | 4 | 69.5 (62.0, 91.0) |  |
| Follow-up minus Baseline | 4 | -8.0 (-17.0, 2.0) | 4 | -3.5 (-14.0, 8.0) | 4 | 2.0 (-7.0, 9.0) | 0.36 |
| P-value: Follow-up vs. Baseline |  | 0.18 |  | 0.56 |  | 0.74 |  |
| 3 minutes |  |  |  |  |  |  |  |
| Baseline | 4 | 86.0 (66.0, 94.0) | 4 | 74.5 (70.0, 97.0) | 4 | 74.5 (70.0, 97.0) |  |
| Follow-up | 4 | 82.0 (75.0, 90.0) | 4 | 70.5 (60.0, 83.0) | 4 | 68.5 (59.0, 90.0) |  |
| Follow-up minus Baseline | 4 | -1.5 (-9.0, 9.0) | 4 | -8.5 (-14.0, -1.0) | 4 | -3.0 (-7.0, 7.0) | 0.30 |
| P-value: Follow-up vs. Baseline |  | 0.86 |  | 0.090 |  | 0.65 |  |
| 6 minutes |  |  |  |  |  |  |  |
| Baseline | 4 | 88.5 (68.0, 106.0) | 4 | 77.0 (64.0, 87.0) | 4 | 71.0 (54.0, 101.0) |  |
| Follow-up | 4 | 86.0 (78.0, 97.0) | 4 | 68.0 (60.0, 87.0) | 4 | 69.0 (63.0, 89.0) |  |
| Follow-up minus Baseline | 4 | -0.5 (-13.0, 10.0) | 4 | -5.5 (-17.0, 6.0) | 4 | -2.0 (-12.0, 9.0) | 0.82 |
| P-value: Follow-up vs. Baseline |  | 0.88 |  | 0.40 |  | 0.72 |  |
| 9 minutes |  |  |  |  |  |  |  |
| Baseline | 4 | 92.5 (66.0, 101.0) | 4 | 76.5 (63.0, 84.0) | 4 | 71.5 (58.0, 102.0) |  |
| Follow-up | 4 | 84.5 (78.0, 98.0) | 4 | 70.0 (64.0, 87.0) | 4 | 70.5 (58.0, 95.0) |  |
| Follow-up minus Baseline | 4 | -2.5 (-15.0, 13.0) | 4 | -3.5 (-10.0, 8.0) | 4 | -5.0 (-8.0, 9.0) | 0.99 |
| P-value: Follow-up vs. Baseline |  | 0.81 |  | 0.66 |  | 0.60 |  |
| 12 minutes |  |  |  |  |  |  |  |
| Baseline | 4 | 91.0 (68.0, 108.0) | 4 | 74.5 (64.0, 80.0) | 4 | 79.0 (59.0, 98.0) |  |
| Follow-up | 4 | 90.5 (79.0, 99.0) | 4 | 69.0 (58.0, 86.0) | 4 | 72.0 (59.0, 89.0) |  |
| Follow-up minus Baseline | 4 | 1.0 (-12.0, 11.0) | 4 | -1.0 (-15.0, 6.0) | 4 | -4.0 (-21.0, 6.0) | 0.75 |
| P-value: Follow-up vs. Baseline |  | 0.97 |  | 0.62 |  | 0.41 |  |
| 15 minutes |  |  |  |  |  |  |  |
| Baseline | 4 | 88.0 (75.0, 103.0) | 4 | 76.5 (64.0, 87.0) | 4 | 69.0 (59.0, 100.0) |  |
| Follow-up | 4 | 87.5 (77.0, 103.0) | 4 | 72.0 (62.0, 93.0) | 4 | 69.5 (58.0, 87.0) |  |
| Follow-up minus Baseline | 4 | -1.5 (-9.0, 13.0) | 4 | 1.5 (-14.0, 6.0) | 4 | -3.0 (-13.0, 6.0) | 0.86 |
| P-value: Follow-up vs. Baseline |  | 0.96 |  | 0.80 |  | 0.47 |  |
| Mean of 0 to 15 minutes |  |  |  |  |  |  |  |
| Baseline | 4 | 88.8 (68.5, 103.2) | 4 | 75.3 (65.3, 84.8) | 4 | 71.0 (57.0, 99.3) |  |
| Follow-up | 4 | 85.5 (76.3, 94.8) | 4 | 69.5 (60.7, 85.7) | 4 | 69.8 (59.8, 90.2) |  |
| Follow-up minus Baseline | 4 | -2.3 (-10.3, 7.8) | 4 | -1.5 (-13.2, 0.8) | 4 | -3.6 (-9.2, 7.7) | 0.93 |
| P-value: Follow-up vs. Baseline |  | 0.74 |  | 0.33 |  | 0.58 |  |
|  |  |  |  |  |  |  |  |
| **Orthostatic MAP** |  |  |  |  |  |  |  |
| 0 minutes |  |  |  |  |  |  |  |
| Baseline | 4 | 96.7 (79.3, 103.7) | 4 | 98.8 (97.3, 113.7) | 4 | 103.3 (93.7, 115.7) |  |
| Follow-up | 4 | 92.2 (84.7, 101.7) | 4 | 95.5 (82.3, 103.7) | 4 | 100.0 (96.3, 119.7) |  |
| Follow-up minus Baseline | 4 | -4.5 (-19.0, 22.3) | 4 | -9.3 (-16.0, 3.0) | 4 | 1.3 (-6.7, 4.0) | 0.59 |
| P-value: Follow-up vs. Baseline |  | 0.88 |  | 0.14 |  | 1.00 |  |
| 3 minutes |  |  |  |  |  |  |  |
| Baseline | 4 | 91.8 (71.3, 108.0) | 4 | 111.7 (88.3, 113.7) | 4 | 98.2 (94.3, 131.0) |  |
| Follow-up | 4 | 96.5 (82.3, 101.0) | 4 | 103.3 (80.3, 106.3) | 4 | 99.2 (94.7, 120.7) |  |
| Follow-up minus Baseline | 4 | -0.2 (-9.7, 23.3) | 4 | -7.8 (-30.7, 14.3) | 4 | -0.2 (-10.3, 2.7) | 0.53 |
| P-value: Follow-up vs. Baseline |  | 0.67 |  | 0.45 |  | 0.55 |  |
| 6 minutes |  |  |  |  |  |  |  |
| Baseline | 4 | 97.3 (84.0, 103.3) | 4 | 101.7 (87.7, 113.3) | 4 | 97.3 (96.3, 131.0) |  |
| Follow-up | 4 | 91.8 (79.0, 94.3) | 4 | 99.0 (85.0, 104.0) | 4 | 98.8 (95.3, 124.3) |  |
| Follow-up minus Baseline | 4 | -7.3 (-11.3, 1.0) | 4 | -4.0 (-21.7, 12.3) | 4 | -1.2 (-6.7, 4.3) | 0.75 |
| P-value: Follow-up vs. Baseline |  | 0.11 |  | 0.59 |  | 0.65 |  |
| 9 minutes |  |  |  |  |  |  |  |
| Baseline | 4 | 96.2 (90.7, 109.0) | 4 | 105.8 (95.0, 114.0) | 4 | 95.8 (94.0, 113.0) |  |
| Follow-up | 4 | 90.7 (83.3, 97.7) | 4 | 93.2 (85.3, 106.0) | 4 | 99.7 (89.7, 108.0) |  |
| Follow-up minus Baseline | 4 | -6.8 (-11.3, -4.7) | 4 | -7.0 (-23.0, -6.0) | 4 | -1.2 (-5.0, 5.7) | 0.086 |
| P-value: Follow-up vs. Baseline |  | 0.014 |  | 0.079 |  | 0.88 |  |
| 12 minutes |  |  |  |  |  |  |  |
| Baseline | 4 | 99.0 (85.7, 108.0) | 4 | 101.5 (90.3, 120.0) | 4 | 98.3 (93.7, 110.0) |  |
| Follow-up | 4 | 98.8 (77.3, 107.0) | 4 | 92.8 (80.7, 104.3) | 4 | 95.5 (94.3, 107.7) |  |
| Follow-up minus Baseline | 4 | -4.5 (-22.0, 21.3) | 4 | -11.3 (-20.0, 0.0) | 4 | -2.3 (-3.3, 0.7) | 0.52 |
| P-value: Follow-up vs. Baseline |  | 0.81 |  | 0.097 |  | 0.12 |  |
| 15 minutes |  |  |  |  |  |  |  |
| Baseline | 4 | 94.2 (86.3, 106.0) | 4 | 101.2 (85.7, 116.3) | 4 | 93.5 (92.7, 120.0) |  |
| Follow-up | 4 | 93.5 (88.7, 99.7) | 4 | 92.0 (82.3, 101.7) | 4 | 103.2 (96.3, 115.7) |  |
| Follow-up minus Baseline | 4 | -0.7 (-6.3, 2.3) | 4 | -8.3 (-21.3, 1.7) | 4 | 4.0 (-4.3, 15.0) | 0.11 |
| P-value: Follow-up vs. Baseline |  | 0.56 |  | 0.19 |  | 0.32 |  |
| Mean of 0 to 15 minutes |  |  |  |  |  |  |  |
| Mean of 0 to 15 minutes |  |  |  |  |  |  |  |
| Baseline | 4 | 93.8 (86.9, 106.3) | 4 | 102.4 (92.7, 115.2) | 4 | 97.3 (94.9, 120.1) |  |
| Follow-up | 4 | 94.9 (85.4, 95.4) | 4 | 96.0 (82.7, 104.3) | 4 | 99.3 (94.7, 116.0) |  |
| Follow-up minus Baseline | 4 | -2.8 (-10.9, 6.2) | 4 | -6.4 (-22.1, 1.1) | 4 | 0.1 (-4.1, 3.3) | 0.32 |
| P-value: Follow-up vs. Baseline |  | 0.52 |  | 0.20 |  | 0.94 |  |
|  |  |  |  |  |  |  |  |
| **Supine MAP** |  |  |  |  |  |  |  |
| 0 minutes |  |  |  |  |  |  |  |
| Baseline | 4 | 95.2 (90.0, 102.3) | 4 | 108.5 (91.0, 116.0) | 4 | 93.8 (87.7, 105.7) |  |
| Follow-up | 4 | 97.8 (95.7, 101.0) | 4 | 95.0 (86.0, 103.3) | 4 | 93.2 (82.7, 107.7) |  |
| Follow-up minus Baseline | 4 | 1.8 (-3.7, 9.7) | 4 | -7.0 (-25.7, -5.0) | 4 | -0.2 (-10.7, 6.7) | 0.092 |
| P-value: Follow-up vs. Baseline |  | 0.50 |  | 0.11 |  | 0.79 |  |
| 3 minutes |  |  |  |  |  |  |  |
| Baseline | 4 | 96.5 (86.3, 106.3) | 4 | 106.2 (97.3, 110.7) | 4 | 91.3 (90.0, 109.0) |  |
| Follow-up | 4 | 96.8 (89.3, 98.0) | 4 | 97.0 (90.0, 104.3) | 4 | 93.0 (84.3, 102.7) |  |
| Follow-up minus Baseline | 4 | 1.3 (-17.0, 9.7) | 4 | -4.5 (-20.7, -2.3) | 4 | -4.0 (-7.7, 7.0) | 0.56 |
| P-value: Follow-up vs. Baseline |  | 0.86 |  | 0.16 |  | 0.56 |  |
| 6 minutes |  |  |  |  |  |  |  |
| Baseline | 4 | 98.8 (76.0, 105.7) | 4 | 102.8 (94.7, 110.3) | 4 | 92.2 (91.3, 103.0) |  |
| Follow-up | 4 | 89.2 (88.7, 97.3) | 4 | 94.7 (82.0, 106.0) | 4 | 93.2 (82.3, 105.3) |  |
| Follow-up minus Baseline | 4 | -5.8 (-16.3, 13.0) | 4 | -7.3 (-19.7, 1.0) | 4 | -0.7 (-10.3, 7.0) | 0.61 |
| P-value: Follow-up vs. Baseline |  | 0.59 |  | 0.18 |  | 0.78 |  |
| Mean of 0 to 6 minutes |  |  |  |  |  |  |  |
| Baseline | 4 | 96.6 (84.6, 104.8) | 4 | 105.8 (94.3, 112.3) | 4 | 92.4 (89.7, 105.9) |  |
| Follow-up | 4 | 94.7 (92.4, 97.3 | 4 | 94.7 (87.7, 104.6) | 4 | 93.1 (83.1, 105.2) |  |
| Follow-up minus Baseline | 4 | -0.9 (-12.3, 10.8) | 4 | -5.4 (-22.0, -3.9) | 4 | -1.6 (-9.6, 6.9) | 0.35 |
| P-value: Follow-up vs. Baseline |  | 0.88 |  | 0.12 |  | 0.69 |  |
| For comparisons of follow-up vs. baseline values within a given treatment group, p-values result from a paired t-test. For comparisons of follow-up minus baseline values between the three treatment groups, p-values result from a one-way ANOVA. | | | | | | | |

# **Supplementary Table 2:** Comparison of baseline patient characteristics between treatment groups

|  | Placebo group (N=4) | | Sitagliptin group (N=4) | | Dapagliflozin group (N=4) | |  |
| --- | --- | --- | --- | --- | --- | --- | --- |
| Variable | N | Median (minimum, maximum) or No. (%) of patients | N | Median (minimum, maximum) or No. (%) of patients | N | Median (minimum, maximum) or No. (%) of patients | P-value |
| Age (years) | 4 | 66.3 (61.7, 75.4) | 4 | 73.9 (61.9, 81.0) | 4 | 69.5 (62.3, 76.9) | 0.58 |
| Sex (Male) | 4 | 2 (50.0%) | 4 | 2 (50.0%) | 4 | 3 (75.0%) | 1.00 |
| Race (White) | 4 | 4 (100.0%) | 4 | 4 (100.0%) | 4 | 3 (75.0%) | 1.00 |
| Ethnicity (Not Hispanic or Latino) | 4 | 4 (100.0%) | 4 | 4 (100.0%) | 4 | 4 (100.0%) | 1.00 |
| Diagnosis | 4 |  | 4 |  | 4 |  | 1.00 |
| Parkinson’s disease |  | 4 (100.0%) |  | 3 (75.0%) |  | 4 (100.0%) |  |
| Lewy body dementia |  | 0 (0.0%) |  | 1 (25.0%) |  | 0 (0.0%) |  |
| Medical History | 4 |  | 4 |  | 4 |  | 1.00 |
| Prediabetes |  | 3 (75.0%) |  | 3 (75.0%) |  | 4 (100.0%) |  |
| Diabetes Mellitus |  | 1 (25.0%) |  | 1 (25.0%) |  | 0 (0.0%) |  |
| Medications |  |  |  |  |  |  |  |
| Metformin | 4 | 1 (25.0%) | 4 | 2 (50.0%) | 4 | 0 (0.0%) | 0.71 |
| Levodopa | 4 | 4 (100.0%) | 4 | 4 (100.0%) | 4 | 4 (100.0%) | 1.00 |
| Amantadine | 4 | 1 (25.0%) | 4 | 1 (25.0%) | 4 | 3 (75.0%) | 0.45 |
| Anticholinergics | 4 | 0 (0.0%) | 4 | 0 (0.0%) | 4 | 0 (0.0%) | 1.00 |
| Pimavanserin | 4 | 0 (0.0%) | 4 | 0 (0.0%) | 4 | 0 (0.0%) | 1.00 |
| COMT | 4 | 0 (0.0%) | 4 | 0 (0.0%) | 4 | 0 (0.0%) | 1.00 |
| Antihypertensive | 4 | 2 (50.0%) | 4 | 2 (50.0%) | 4 | 0 (0.0%) | 0.42 |
| None | 4 | 0 (0.0%) | 4 | 0 (0.0%) | 4 | 0 (0.0%) | 1.00 |
| Vitals information |  |  |  |  |  |  |  |
| Systolic blood pressure at baseline | 4 | 127.2 (115.0, 145.0) | 4 | 144.5 (132.3, 163.7) | 4 | 120.7 (115.7, 143.0) | 0.11 |
| Diastolic blood pressure at baseline | 4 | 81.0 (69.3, 85.3) | 4 | 86.0 (75.3, 87.7) | 4 | 78.3 (76.7, 87.3) | 0.56 |
| Heart rate at baseline | 4 | 70.8 (66.7, 74.7) | 4 | 65.7 (53.7, 68.3) | 4 | 65.2 (53.7, 81.0) | 0.49 |
| Lab values |  |  |  |  |  |  |  |
| Leukocytes | 4 | 8.2 (6.5, 8.2) | 4 | 6.5 (5.5, 7.2) | 4 | 5.7 (3.7, 8.5) | 0.21 |
| Erythrocytes | 4 | 5.1 (4.5, 5.5) | 4 | 5.1 (4.1, 5.5) | 4 | 4.5 (4.5, 5.0) | 0.44 |
| Hemoglobin | 4 | 14.5 (13.3, 16.2) | 4 | 16.1 (12.0, 17.4) | 4 | 14.6 (14.0, 15.3) | 0.74 |
| Hematocrit | 4 | 43.0 (41.0, 47.0) | 4 | 47.0 (38.0, 49.0) | 4 | 43.5 (40.0, 46.0) | 0.71 |
| MCV | 4 | 90.0 (77.3, 93.0) | 4 | 91.7 (89.8, 92.5) | 4 | 93.9 (90.8, 97.6) | 0.19 |
| MCH | 4 | 30.3 (25.1, 31.4) | 4 | 30.9 (29.0, 32.5) | 4 | 31.6 (30.7, 32.6) | 0.28 |
| MCHC | 4 | 33.5 (32.5, 34.0) | 4 | 34.1 (31.5, 35.2) | 4 | 33.6 (32.7, 34.4) | 0.91 |
| RDW CV | 4 | 13.4 (12.9, 14.6) | 4 | 12.8 (12.2, 13.8) | 4 | 12.2 (11.4, 13.9) | 0.21 |
| RDW SD | 4 | 43.4 (40.5, 45.9) | 4 | 43.2 (40.1, 47.2) | 3 | 44.9 (39.1, 48.1) | 0.95 |
| Platelet count | 4 | 217.5 (201.0, 314.0) | 4 | 202.0 (165.0, 235.0) | 4 | 219.5 (185.0, 294.0) | 0.50 |
| Mean platelet volume | 4 | 10.7 (9.9, 11.4) | 4 | 10.3 (9.7, 12.9) | 4 | 10.5 (9.2, 10.8) | 0.73 |
| Neutrophils | 4 | 64.5 (4.6, 74.5) | 4 | 64.6 (54.0, 69.4) | 4 | 59.2 (53.3, 2195.0) | 0.41 |
| Immature granulocytes | 4 | 0.3 (0.2, 0.5) | 4 | 0.5 (0.1, 0.9) | 3 | 0.4 (0.3, 0.5) | 0.66 |
| Lymphocytes | 4 | 18.2 (0.4, 31.5) | 4 | 24.9 (21.6, 35.6) | 4 | 31.7 (26.1, 42.1) | 0.097 |
| Monocytes | 4 | 7.3 (6.3, 9.2) | 4 | 7.0 (7.0, 8.6) | 4 | 9.0 (6.9, 456.0) | 0.40 |
| Eosinophils | 4 | 1.6 (0.2, 2.1) | 4 | 1.7 (0.7, 2.7) | 4 | 3.8 (2.8, 52.0) | 0.31 |
| Basophils | 4 | 0.9 (0.5, 1.0) | 4 | 0.6 (0.4, 0.8) | 4 | 1.0 (0.0, 19.0) | 0.41 |
| Hemoglobin A1C | 4 | 6.2 (5.8, 6.8) | 4 | 6.7 (5.7, 7.2) | 4 | 5.7 (4.7, 6.2) | 0.11 |
| Estimated average glucose | 4 | 130.0 (120.0, 148.0) | 4 | 145.5 (117.0, 160.0) | 4 | 115.5 (78.0, 131.0) | 0.11 |
| Potassium | 4 | 4.5 (3.7, 4.6) | 4 | 4.2 (4.2, 4.5) | 4 | 4.2 (3.7, 4.6) | 0.80 |
| Sodium | 4 | 139.5 (136.0, 142.0) | 4 | 139.5 (139.0, 142.0) | 4 | 138.5 (138.0, 143.0) | 0.88 |
| Chloride | 4 | 104.0 (101.0, 107.0) | 4 | 103.0 (102.0, 105.0) | 4 | 102.0 (101.0, 103.0) | 0.32 |
| Bicarbonate | 4 | 25.0 (24.0, 25.0) | 4 | 25.0 (21.0, 29.0) | 3 | 24.0 (24.0, 27.0) | 0.98 |
| Anion gap | 4 | 10.5 (8.0, 13.0) | 4 | 12.0 (10.0, 13.0) | 3 | 11.0 (11.0, 11.0) | 0.58 |
| BUN | 4 | 14.0 (11.0, 22.0) | 4 | 22.0 (12.0, 25.0) | 4 | 18.0 (14.0, 21.0) | 0.35 |
| Creatinine | 4 | 0.9 (0.9, 1.0) | 4 | 0.8 (0.5, 1.0) | 4 | 0.9 (0.9, 1.1) | 0.31 |
| Estimated GFR | 4 | 82.5 (73.0, 90.0) | 4 | 87.5 (63.0, 90.0) | 4 | 82.0 (65.0, 90.0) | 0.95 |
| Total calcium | 4 | 9.2 (8.5, 9.7) | 4 | 9.5 (9.4, 9.8) | 4 | 9.3 (8.9, 9.6) | 0.34 |
| Glucose | 4 | 112.5 (95.0, 158.0) | 4 | 139.5 (97.0, 215.0) | 4 | 104.0 (78.0, 131.0) | 0.30 |
| Total protein | 4 | 6.9 (6.4, 7.6) | 4 | 7.1 (6.8, 7.6) | 4 | 7.3 (7.2, 7.4) | 0.44 |
| Albumin | 4 | 4.5 (3.5, 4.7) | 4 | 4.5 (3.8, 4.9) | 4 | 4.4 (4.3, 4.6) | 0.91 |
| Aspartate aminotransferase | 4 | 22.5 (19.0, 24.0) | 4 | 17.0 (14.0, 32.0) | 4 | 30.0 (20.0, 52.0) | 0.16 |
| Alkaline phosphatase | 4 | 88.0 (79.0, 94.0) | 4 | 71.5 (67.0, 89.0) | 3 | 60.0 (60.0, 80.0) | 0.048 |
| Alanine transaminase | 4 | 19.5 (10.0, 38.0) | 4 | 14.5 (8.0, 17.0) | 4 | 19.5 (11.0, 31.0) | 0.48 |
| Total bilirubin | 4 | 0.4 (0.2, 0.9) | 4 | 0.6 (0.4, 1.5) | 3 | 0.6 (0.5, 0.8) | 0.49 |
| Triglycerides | 4 | 131.0 (102.0, 181.0) | 4 | 113.0 (76.0, 315.0) | 4 | 95.5 (53.0, 125.0) | 0.45 |
| Total cholesterol | 4 | 153.0 (123.0, 233.0) | 4 | 138.5 (87.0, 216.0) | 4 | 145.5 (117.0, 190.0) | 0.81 |
| LDL cholesterol | 4 | 83.5 (70.0, 115.0) | 4 | 69.5 (30.0, 125.0) | 4 | 77.5 (60.0, 128.0) | 0.79 |
| HDL cholesterol | 4 | 46.0 (34.0, 87.0) | 4 | 37.0 (28.0, 78.0) | 4 | 46.5 (39.0, 52.0) | 0.81 |
| Non-HDL cholesterol | 4 | 107.0 (89.0, 146.0) | 4 | 106.5 (49.0, 138.0) | 4 | 93.5 (76.0, 151.0) | 0.88 |
| Fasting (8 hours or more) | 4 | 2 (50.0%) | 4 | 2 (50.0%) | 4 | 4 (100.0%) | 0.42 |
| P-values result from a one-way ANOVA (continuous variables) or Fisher’s exact test (categorical variables). | | | | | | | |

**Supplemental Table 3:** Comparison of baseline patient characteristics between treatment groups when excluding the 1 Lewy body dementia patient from Group B

|  | Placebo group (N=4) | | Sitagliptin group (N=3) | | Dapagliflozin group (N=4) | |  |
| --- | --- | --- | --- | --- | --- | --- | --- |
| Variable | N | Median (minimum, maximum) or No. (%) of patients | N | Median (minimum, maximum) or No. (%) of patients | N | Median (minimum, maximum) or No. (%) of patients | P-value |
| Age (years) | 4 | 66.3 (61.7, 75.4) | 3 | 70.5 (61.9, 81.0) | 4 | 69.5 (62.3, 76.9) | 0.79 |
| Sex (Male) | 4 | 2 (50.0%) | 3 | 2 (66.7%) | 4 | 3 (75.0%) | 1.00 |
| Race (White) | 4 | 4 (100.0%) | 3 | 3 (100.0%) | 4 | 3 (75.0%) | 1.00 |
| Ethnicity (Not Hispanic or Latino) | 4 | 4 (100.0%) | 3 | 3 (100.0%) | 4 | 4 (100.0%) | 1.00 |
| Diagnosis | 4 |  | 3 |  | 4 |  | 1.00 |
| Parkinson’s disease |  | 4 (100.0%) |  | 3 (100.0%) |  | 4 (100.0%) |  |
| Lewy body dementia |  | 0 (0.0%) |  | 0 (0.0%) |  | 0 (0.0%) |  |
| Medical History | 4 |  | 3 |  | 4 |  | 0.71 |
| Prediabetes |  | 3 (75.0%) |  | 2 (66.7%) |  | 4 (100.0%) |  |
| Diabetes Mellitus |  | 1 (25.0%) |  | 1 (33.3%) |  | 0 (0.0%) |  |
| Medications |  |  |  |  |  |  |  |
| Metformin | 4 | 1 (25.0%) | 3 | 2 (66.7%) | 4 | 0 (0.0%) | 0.20 |
| Levodopa | 4 | 4 (100.0%) | 3 | 3 (100.0%) | 4 | 4 (100.0%) | 1.00 |
| Amantadine | 4 | 1 (25.0%) | 3 | 1 (33.3%) | 4 | 3 (75.0%) | 0.45 |
| Anticholinergics | 4 | 0 (0.0%) | 3 | 0 (0.0%) | 4 | 0 (0.0%) | 1.00 |
| Pimavanserin | 4 | 0 (0.0%) | 3 | 0 (0.0%) | 4 | 0 (0.0%) | 1.00 |
| COMT | 4 | 0 (0.0%) | 3 | 0 (0.0%) | 4 | 0 (0.0%) | 1.00 |
| Antihypertensive | 4 | 2 (50.0%) | 3 | 1 (33.3%) | 4 | 0 (0.0%) | 0.42 |
| None | 4 | 0 (0.0%) | 3 | 0 (0.0%) | 4 | 0 (0.0%) | 1.00 |
| Vitals information |  |  |  |  |  |  |  |
| Systolic blood pressure at baseline (0 minutes) | 4 | 128.5 (118.0, 137.0) | 3 | 155.0 (144.0, 170.0) | 4 | 122.5 (113.0, 143.0) | 0.014 |
| Systolic blood pressure at baseline (3 minutes) | 4 | 125.5 (117.0, 153.0) | 3 | 152.0 (135.0, 166.0) | 4 | 119.0 (116.0, 147.0) | 0.15 |
| Systolic blood pressure at baseline (6 minutes) | 4 | 131.5 (102.0, 145.0) | 3 | 145.0 (136.0, 155.0) | 4 | 120.5 (118.0, 139.0) | 0.20 |
| Systolic blood pressure at baseline (mean of 0, 3, 6 minutes) | 4 | 127.2 (115.0, 145.0) | 3 | 150.7 (138.3, 163.7) | 4 | 120.7 (115.7, 143.0) | 0.070 |
| Diastolic blood pressure at baseline (0 minutes) | 4 | 79.5 (74.0, 85.0) | 3 | 89.0 (86.0, 90.0) | 4 | 79.5 (75.0, 87.0) | 0.072 |
| Diastolic blood pressure at baseline (3 minutes) | 4 | 80.5 (71.0, 86.0) | 3 | 87.0 (83.0, 88.0) | 4 | 77.5 (77.0, 90.0) | 0.34 |
| Diastolic blood pressure at baseline (6 minutes) | 4 | 82.0 (63.0, 87.0) | 3 | 85.0 (83.0, 88.0) | 4 | 78.5 (77.0, 85.0) | 0.47 |
| Diastolic blood pressure at baseline (mean of 0, 3, 6 minutes) | 4 | 81.0 (69.3, 85.3) | 3 | 86.7 (85.3, 87.7) | 4 | 78.3 (76.7, 87.3) | 0.23 |
| Heart rate at baseline (0 minutes) | 4 | 73.0 (67.0, 75.0) | 3 | 69.0 (50.0, 69.0) | 4 | 64.0 (52.0, 84.0) | 0.52 |
| Heart rate at baseline (3 minutes) | 4 | 68.5 (66.0, 76.0) | 3 | 67.0 (58.0, 68.0) | 4 | 66.0 (55.0, 83.0) | 0.73 |
| Heart rate at baseline (6 minutes) | 4 | 70.5 (67.0, 74.0) | 3 | 66.0 (53.0, 69.0) | 4 | 65.5 (54.0, 76.0) | 0.45 |
| Heart rate at baseline (mean of 0, 3, 6 minutes) | 4 | 70.8 (66.7, 74.7) | 3 | 67.7 (53.7, 68.3) | 4 | 65.2 (53.7, 81.0) | 0.56 |
| Lab values |  |  |  |  |  |  |  |
| Leukocytes | 4 | 8.2 (6.5, 8.2) | 3 | 7.0 (6.0, 7.2) | 4 | 5.7 (3.7, 8.5) | 0.24 |
| Erythrocytes | 4 | 5.1 (4.5, 5.5) | 3 | 5.2 (5.1, 5.5) | 4 | 4.5 (4.5, 5.0) | 0.10 |
| Hemoglobin | 4 | 14.5 (13.3, 16.2) | 3 | 16.5 (15.6, 17.4) | 4 | 14.6 (14.0, 15.3) | 0.055 |
| Hematocrit | 4 | 43.0 (41.0, 47.0) | 3 | 47.0 (47.0, 49.0) | 4 | 43.5 (40.0, 46.0) | 0.089 |
| MCV | 4 | 90.0 (77.3, 93.0) | 3 | 91.3 (89.8, 92.5) | 4 | 93.9 (90.8, 97.6) | 0.23 |
| MCH | 4 | 30.3 (25.1, 31.4) | 3 | 31.6 (30.2, 32.5) | 4 | 31.6 (30.7, 32.6) | 0.23 |
| MCHC | 4 | 33.5 (32.5, 34.0) | 3 | 35.1 (33.1, 35.2) | 4 | 33.6 (32.7, 34.4) | 0.26 |
| RDW CV | 4 | 13.4 (12.9, 14.6) | 3 | 12.4 (12.2, 13.1) | 4 | 12.2 (11.4, 13.9) | 0.17 |
| RDW SD | 4 | 43.4 (40.5, 45.9) | 3 | 42.3 (40.1, 44.1) | 3 | 44.9 (39.1, 48.1) | 0.76 |
| Platelet count | 4 | 217.5 (201.0, 314.0) | 3 | 207.0 (197.0, 235.0) | 4 | 219.5 (185.0, 294.0) | 0.77 |
| Mean platelet volume | 4 | 10.7 (9.9, 11.4) | 3 | 10.1 (9.7, 10.5) | 4 | 10.5 (9.2, 10.8) | 0.50 |
| Neutrophils | 4 | 64.5 (4.6, 74.5) | 3 | 63.2 (54.0, 69.4) | 4 | 59.2 (53.3, 2195.0) | 0.46 |
| Immature granulocytes | 4 | 0.3 (0.2, 0.5) | 3 | 0.5 (0.1, 0.9) | 3 | 0.4 (0.3, 0.5) | 0.65 |
| Lymphocytes | 4 | 18.2 (0.4, 31.5) | 3 | 24.2 (21.6, 35.6) | 4 | 31.7 (26.1, 42.1) | 0.12 |
| Monocytes | 4 | 7.3 (6.3, 9.2) | 3 | 7.0 (7.0, 8.6) | 4 | 9.0 (6.9, 456.0) | 0.46 |
| Eosinophils | 4 | 1.6 (0.2, 2.1) | 3 | 2.5 (0.8, 2.7) | 4 | 3.8 (2.8, 52.0) | 0.37 |
| Basophils | 4 | 0.9 (0.5, 1.0) | 3 | 0.7 (0.4, 0.8) | 4 | 1.0 (0.0, 19.0) | 0.48 |
| Hemoglobin A1C | 4 | 6.2 (5.8, 6.8) | 3 | 7.1 (6.3, 7.2) | 4 | 5.7 (4.7, 6.2) | 0.036 |
| Estimated average glucose | 4 | 130.0 (120.0, 148.0) | 3 | 157.0 (134.0, 160.0) | 4 | 115.5 (78.0, 131.0) | 0.047 |
| Potassium | 4 | 4.5 (3.7, 4.6) | 3 | 4.2 (4.2, 4.5) | 4 | 4.2 (3.7, 4.6) | 0.80 |
| Sodium | 4 | 139.5 (136.0, 142.0) | 3 | 139.0 (139.0, 140.0) | 4 | 138.5 (138.0, 143.0) | 0.99 |
| Chloride | 4 | 104.0 (101.0, 107.0) | 3 | 103.0 (102.0, 105.0) | 4 | 102.0 (101.0, 103.0) | 0.36 |
| Bicarbonate | 4 | 25.0 (24.0, 25.0) | 3 | 24.0 (21.0, 26.0) | 3 | 24.0 (24.0, 27.0) | 0.60 |
| Anion gap | 4 | 10.5 (8.0, 13.0) | 3 | 12.0 (12.0, 13.0) | 3 | 11.0 (11.0, 11.0) | 0.36 |
| BUN | 4 | 14.0 (11.0, 22.0) | 3 | 22.0 (12.0, 22.0) | 4 | 18.0 (14.0, 21.0) | 0.59 |
| Creatinine | 4 | 0.9 (0.9, 1.0) | 3 | 0.9 (0.8, 1.0) | 4 | 0.9 (0.9, 1.1) | 0.53 |
| Estimated GFR | 4 | 82.5 (73.0, 90.0) | 3 | 85.0 (63.0, 90.0) | 4 | 82.0 (65.0, 90.0) | 0.94 |
| Total calcium | 4 | 9.2 (8.5, 9.7) | 3 | 9.5 (9.4, 9.8) | 4 | 9.3 (8.9, 9.6) | 0.37 |
| Glucose | 4 | 112.5 (95.0, 158.0) | 3 | 163.0 (116.0, 215.0) | 4 | 104.0 (78.0, 131.0) | 0.11 |
| Total protein | 4 | 6.9 (6.4, 7.6) | 3 | 7.0 (6.8, 7.6) | 4 | 7.3 (7.2, 7.4) | 0.48 |
| Albumin | 4 | 4.5 (3.5, 4.7) | 3 | 4.5 (4.5, 4.9) | 4 | 4.4 (4.3, 4.6) | 0.51 |
| Aspartate aminotransferase | 4 | 22.5 (19.0, 24.0) | 3 | 18.0 (16.0, 32.0) | 4 | 30.0 (20.0, 52.0) | 0.25 |
| Alkaline phosphatase | 4 | 88.0 (79.0, 94.0) | 3 | 73.0 (70.0, 89.0) | 3 | 60.0 (60.0, 80.0) | 0.060 |
| Alanine transaminase | 4 | 19.5 (10.0, 38.0) | 3 | 14.0 (8.0, 17.0) | 4 | 19.5 (11.0, 31.0) | 0.54 |
| Total bilirubin | 4 | 0.4 (0.2, 0.9) | 3 | 0.7 (0.4, 1.5) | 3 | 0.6 (0.5, 0.8) | 0.40 |
| Triglycerides | 4 | 131.0 (102.0, 181.0) | 3 | 124.0 (102.0, 315.0) | 4 | 95.5 (53.0, 125.0) | 0.26 |
| Total cholesterol | 4 | 153.0 (123.0, 233.0) | 3 | 112.0 (87.0, 165.0) | 4 | 145.5 (117.0, 190.0) | 0.40 |
| LDL cholesterol | 4 | 83.5 (70.0, 115.0) | 3 | 61.0 (30.0, 78.0) | 4 | 77.5 (60.0, 128.0) | 0.29 |
| HDL cholesterol | 4 | 46.0 (34.0, 87.0) | 3 | 36.0 (28.0, 38.0) | 4 | 46.5 (39.0, 52.0) | 0.31 |
| Non-HDL cholesterol | 4 | 107.0 (89.0, 146.0) | 3 | 84.0 (49.0, 129.0) | 4 | 93.5 (76.0, 151.0) | 0.63 |
| Fasting (8 hours or more) | 4 | 2 (50.0%) | 3 | 2 (66.7%) | 4 | 4 (100.0%) | 0.18 |
| P-values result from a one-way ANOVA (continuous variables) or Fisher’s exact test (categorical variables). | | | | | | | |

# **Supplemental Table 4:** Comparisons of changes in MDS-UPDRS and MMSE from baseline to follow-up within and between treatment groups when excluding the 1 Lewy body dementia patient from Group B

|  | Placebo group (N=4) | | Sitagliptin group (N=3) | | Dapagliflozin group (N=4) | |  |
| --- | --- | --- | --- | --- | --- | --- | --- |
| Outcome | N | Median (minimum, maximum) or p-value | N | Median (minimum, maximum) or p-value | N | Median (minimum, maximum) or p-value | P-value for comparison between groups of change between time points |
| MDS-UPDRS score – part 1 |  |  |  |  |  |  |  |
| Baseline | 4 | 8.0 (2.0, 22.0) | 3 | 5.0 (2.0, 10.0) | 4 | 7.0 (6.0, 9.0) |  |
| Follow-up | 4 | 5.5 (1.0, 18.0) | 3 | 6.0 (4.0, 14.0) | 4 | 4.0 (2.0, 7.0) |  |
| Follow-up minus Baseline | 4 | -2.5 (-4.0, -1.0) | 3 | 2.0 (1.0, 4.0) | 4 | -2.5 (-7.0, 0.0) | 0.029 |
| P-value: Follow-up vs. Baseline |  | 0.063 |  | 0.11 |  | 0.13 |  |
| MDS-UPDRS score – part 2 |  |  |  |  |  |  |  |
| Baseline | 4 | 6.5 (1.0, 23.0) | 3 | 8.0 (4.0, 12.0) | 4 | 9.0 (4.0, 19.0) |  |
| Follow-up | 4 | 7.0 (0.0, 20.0) | 3 | 9.0 (4.0, 16.0) | 4 | 6.5 (3.0, 15.0) |  |
| Follow-up minus Baseline | 4 | -0.5 (-3.0, 1.0) | 3 | 1.0 (0.0, 4.0) | 4 | -3.0 (-4.0, 0.0) | 0.049 |
| P-value: Follow-up vs. Baseline |  | 0.44 |  | 0.30 |  | 0.063 |  |
| MDS-UPDRS score – part 3 |  |  |  |  |  |  |  |
| Baseline | 4 | 25.5 (18.0, 39.0) | 3 | 30.0 (19.0, 37.0) | 4 | 38.0 (24.0, 50.0) |  |
| Follow-up | 4 | 25.5 (18.0, 41.0) | 3 | 24.0 (18.0, 37.0) | 4 | 36.5 (10.0, 47.0) |  |
| Follow-up minus Baseline | 4 | 0.0 (0.0, 2.0) | 3 | -1.0 (-6.0, 0.0) | 4 | -3.0 (-14.0, 0.0) | 0.23 |
| P-value: Follow-up vs. Baseline |  | 0.39 |  | 0.34 |  | 0.20 |  |
| MDS-UPDRS score – part 4 |  |  |  |  |  |  |  |
| Baseline | 4 | 0.5 (0.0, 2.0) | 3 | 0.0 (0.0, 0.0) | 4 | 0.0 (0.0, 4.0) |  |
| Follow-up | 4 | 0.0 (0.0, 1.0) | 3 | 0.0 (0.0, 8.0) | 4 | 0.0 (0.0, 1.0) |  |
| Follow-up minus Baseline | 4 | 0.0 (-2.0, 0.0) | 3 | 0.0 (0.0, 8.0) | 4 | 0.0 (-4.0, 1.0) | 0.26 |
| P-value: Follow-up vs. Baseline |  | 0.39 |  | 0.42 |  | 0.55 |  |
| MDS-UPDRS total score |  |  |  |  |  |  |  |
| Baseline | 4 | 49.5 (22.0, 67.0) | 3 | 43.0 (32.0, 52.0) | 4 | 52.5 (43.0, 76.0) |  |
| Follow-up | 4 | 47.5 (20.0, 60.0) | 3 | 45.0 (33.0, 62.0) | 4 | 48.5 (15.0, 67.0) |  |
| Follow-up minus Baseline | 4 | -3.5 (-7.0, 1.0) | 3 | 2.0 (1.0, 10.0) | 4 | -7.0 (-28.0, -3.0) | 0.080 |
| P-value: Follow-up vs. Baseline |  | 0.16 |  | 0.27 |  | 0.14 |  |
| MMSE score |  |  |  |  |  |  |  |
| Baseline | 4 | 26.5 (23.0, 29.0) | 3 | 26.0 (23.0, 26.0) | 4 | 28.0 (25.0, 30.0) |  |
| Follow-up | 4 | 28.0 (24.0, 30.0) | 3 | 26.0 (24.0, 30.0) | 4 | 28.0 (28.0, 29.0) |  |
| Follow-up minus Baseline | 4 | 1.5 (-1.0, 3.0) | 3 | 1.0 (0.0, 4.0) | 4 | 0.5 (-2.0, 3.0) | 0.76 |
| P-value: Follow-up vs. Baseline |  | 0.24 |  | 0.30 |  | 0.70 |  |
| For comparisons of follow-up vs. baseline values within a given treatment group, p-values result from a paired t-test. For comparisons of follow-up minus baseline values between the three treatment groups, p-values result from a one-way ANOVA. | | | | | | | |

# **Supplemental Table 5:** Comparisons of changes in orthostatic systolic blood pressure, orthostatic diastolic blood pressure, orthostatic heart rate, orthostatic MAP, and supine MAP from baseline to follow-up within and between treatment groups when excluding the 1 Lewy body dementia patient from Group B

|  | Placebo group (N=4) | | Sitagliptin group (N=3) | | Dapagliflozin group (N=4) | |  |
| --- | --- | --- | --- | --- | --- | --- | --- |
| Outcome | N | Median (minimum, maximum) or p-value | N | Median (minimum, maximum) or p-value | N | Median (minimum, maximum) or p-value | P-value for comparison between groups of change between time points |
| **Orthostatic systolic blood pressure (SBP)** |  |  |  |  |  |  |  |
| 0 minutes |  |  |  |  |  |  |  |
| Baseline | 4 | 126.0 (100.0, 145.0) | 3 | 147.0 (130.0, 159.0) | 4 | 141.0 (121.0, 161.0) |  |
| Follow-up | 4 | 124.5 (106.0, 143.0) | 3 | 127.0 (109.0, 145.0) | 4 | 131.5 (124.0, 155.0) |  |
| Follow-up minus Baseline | 4 | -6.5 (-29.0, 43.0) | 3 | -14.0 (-38.0, -3.0) | 4 | -5.0 (-18.0, 6.0) | 0.55 |
| P-value: Follow-up vs. Baseline |  | 0.99 |  | 0.22 |  | 0.35 |  |
| 3 minutes |  |  |  |  |  |  |  |
| Baseline | 4 | 124.0 (102.0, 139.0) | 3 | 153.0 (143.0, 155.0) | 4 | 128.5 (123.0, 181.0 |  |
| Follow-up | 4 | 123.0 (122.0, 133.0) | 3 | 136.0 (107.0, 145.0) | 4 | 128.5 (118.0, 166.0) |  |
| Follow-up minus Baseline | 4 | 3.0 (-15.0, 21.0) | 3 | -10.0 (-46.0, -7.0) | 4 | -3.5 (-15.0, 2.0) | 0.19 |
| P-value: Follow-up vs. Baseline |  | 0.74 |  | 0.24 |  | 0.26 |  |
| 6 minutes |  |  |  |  |  |  |  |
| Baseline | 4 | 133.0 (108.0, 138.0) | 3 | 154.0 (124.0, 156.0) | 4 | 128.0 (123.0, 177.0) |  |
| Follow-up | 4 | 120.5 (107.0, 133.0) | 3 | 124.0 (113.0, 138.0) | 4 | 129.0 (121.0, 163.0) |  |
| Follow-up minus Baseline | 4 | -7.5 (-15.0, -1.0) | 3 | -18.0 (-41.0, 0.0) | 4 | -2.5 (-14.0, 5.0) | 0.26 |
| P-value: Follow-up vs. Baseline |  | 0.084 |  | 0.24 |  | 0.46 |  |
| 9 minutes |  |  |  |  |  |  |  |
| Baseline | 4 | 124.5 (118.0, 137.0) | 3 | 149.0 (136.0, 158.0) | 4 | 124.5 (118.0, 155.0) |  |
| Follow-up | 4 | 116.5 (115.0, 123.0) | 3 | 122.0 (110.0, 144.0) | 4 | 131.0 (117.0, 144.0) |  |
| Follow-up minus Baseline | 4 | -8.0 (-14.0, -3.0) | 3 | -14.0 (-39.0, -14.0) | 4 | 1.5 (-11.0, 9.0) | 0.042 |
| P-value: Follow-up vs. Baseline |  | 0.059 |  | 0.16 |  | 0.96 |  |
| 12 minutes |  |  |  |  |  |  |  |
| Baseline | 4 | 128.0 (107.0, 140.0) | 3 | 134.0 (113.0, 162.0) | 4 | 130.0 (121.0, 146.0) |  |
| Follow-up | 4 | 128.5 (106.0, 139.0) | 3 | 115.0 (106.0, 145.0) | 4 | 122.5 (117.0, 147.0) |  |
| Follow-up minus Baseline | 4 | -6.5 (-20.0, 32.0) | 3 | -17.0 (-28.0, 2.0) | 4 | -5.5 (-8.0, 1.0) | 0.58 |
| P-value: Follow-up vs. Baseline |  | 0.99 |  | 0.24 |  | 0.11 |  |
| 15 minutes |  |  |  |  |  |  |  |
| Baseline | 4 | 133.5 (117.0, 146.0) | 3 | 139.0 (126.0, 153.0) | 4 | 122.0 (117.0, 152.0) |  |
| Follow-up | 4 | 119.5 (116.0, 121.0) | 3 | 120.0 (105.0, 139.0) | 4 | 134.0 (124.0, 155.0) |  |
| Follow-up minus Baseline | 4 | -14.0 (-25.0, -1.0) | 3 | -14.0 (-34.0, -6.0) | 4 | 5.0 (3.0, 21.0) | 0.027 |
| P-value: Follow-up vs. Baseline |  | 0.098 |  | 0.16 |  | 0.14 |  |
| Mean of 0 to 15 minutes |  |  |  |  |  |  |  |
| Baseline | 4 | 128.0 (112.2, 137.7) | 3 | 146.0 (128.7, 157.2) | 4 | 129.0 (120.5, 162.0) |  |
| Follow-up | 4 | 121.0 (118.5, 127.7) | 3 | 124.0 (108.3, 142.7) | 4 | 128.9 (121.2, 155.0) |  |
| Follow-up minus Baseline | 4 | -2.5 (-19.0, 6.3) | 3 | -14.5 (-37.7, -4.7) | 4 | -1.1 (-7.0, 2.7) | 0.18 |
| P-value: Follow-up vs. Baseline |  | 0.51 |  | 0.19 |  | 0.50 |  |
|  |  |  |  |  |  |  |  |
| **Orthostatic diastolic blood pressure (DBP)** |  |  |  |  |  |  |  |
| 0 minutes |  |  |  |  |  |  |  |
| Baseline | 4 | 79.5 (69.0, 88.0) | 3 | 84.0 (74.0, 91.0) | 4 | 84.5 (80.0, 93.0) |  |
| Follow-up | 4 | 76.5 (73.0, 81.0) | 3 | 83.0 (69.0, 90.0) | 4 | 85.0 (81.0, 102.0) |  |
| Follow-up minus Baseline | 4 | -3.5 (-14.0, 12.0) | 3 | -5.0 (-8.0, 6.0) | 4 | 1.5 (-1.0, 9.0) | 0.62 |
| P-value: Follow-up vs. Baseline |  | 0.70 |  | 0.64 |  | 0.30 |  |
| 3 minutes |  |  |  |  |  |  |  |
| Baseline | 4 | 77.5 (52.0, 93.0) | 3 | 93.0 (90.0, 97.0) | 4 | 83.0 (80.0, 106.0) |  |
| Follow-up | 4 | 83.0 (62.0, 86.0) | 3 | 87.0 (67.0, 88.0) | 4 | 84.5 (83.0, 98.0) |  |
| Follow-up minus Baseline | 4 | -2.5 (-10.0, 29.0) | 3 | -9.0 (-23.0, -6.0) | 4 | 1.0 (-8.0, 4.0) | 0.27 |
| P-value: Follow-up vs. Baseline |  | 0.72 |  | 0.14 |  | 0.87 |  |
| 6 minutes |  |  |  |  |  |  |  |
| Baseline | 4 | 79.0 (71.0, 88.0) | 3 | 83.0 (83.0, 92.0) | 4 | 83.0 (81.0, 108.0) |  |
| Follow-up | 4 | 74.5 (65.0, 81.0) | 3 | 85.0 (71.0, 87.0) | 4 | 84.5 (81.0, 105.0) |  |
| Follow-up minus Baseline | 4 | -7.0 (-12.0, 4.0) | 3 | -5.0 (-12.0, 2.0) | 4 | 0.0 (-4.0, 4.0) | 0.41 |
| P-value: Follow-up vs. Baseline |  | 0.20 |  | 0.34 |  | 1.00 |  |
| 9 minutes |  |  |  |  |  |  |  |
| Baseline | 4 | 82.5 (76.0, 95.0) | 3 | 88.0 (87.0, 92.0) | 4 | 82.0 (81.0, 92.0) |  |
| Follow-up | 4 | 78.0 (67.0, 85.0) | 3 | 85.0 (73.0, 87.0) | 4 | 84.0 (76.0, 90.0) |  |
| Follow-up minus Baseline | 4 | -8.5 (-10.0, -1.0) | 3 | -5.0 (-15.0, -2.0) | 4 | -0.5 (-6.0, 4.0) | 0.18 |
| P-value: Follow-up vs. Baseline |  | 0.042 |  | 0.20 |  | 0.75 |  |
| 12 minutes |  |  |  |  |  |  |  |
| Baseline | 4 | 84.5 (75.0, 92.0) | 3 | 84.0 (79.0, 99.0) | 4 | 82.5 (80.0, 92.0) |  |
| Follow-up | 4 | 84.0 (63.0, 91.0) | 3 | 78.0 (68.0, 84.0) | 4 | 83.0 (81.0, 88.0) |  |
| Follow-up minus Baseline | 4 | -3.5 (-23.0, 16.0) | 3 | -15.0 (-16.0, -1.0) | 4 | -0.5 (-4.0, 3.0) | 0.49 |
| P-value: Follow-up vs. Baseline |  | 0.69 |  | 0.16 |  | 0.75 |  |
| 15 minutes |  |  |  |  |  |  |  |
| Baseline | 4 | 73.5 (71.0, 88.0) | 3 | 86.0 (85.0, 98.0) | 4 | 80.5 (78.0, 104.0) |  |
| Follow-up | 4 | 80.5 (75.0, 89.0) | 3 | 83.0 (71.0, 85.0) | 4 | 88.0 (82.0, 96.0) |  |
| Follow-up minus Baseline | 4 | 5.0 (1.0, 8.0) | 3 | -15.0 (-15.0, 0.0) | 4 | 3.5 (-8.0, 12.0) | 0.051 |
| P-value: Follow-up vs. Baseline |  | 0.050 |  | 0.18 |  | 0.55 |  |
| Mean of 0 to 15 minutes |  |  |  |  |  |  |  |
| Baseline | 4 | 77.5 (72.8, 90.7) | 3 | 85.8 (84.2, 94.2) | 4 | 82.2 (80.8, 99.2) |  |
| Follow-up | 4 | 79.6 (68.8, 83.8) | 3 | 85.2 (69.8, 85.2) | 4 | 84.4 (81.5, 96.5) |  |
| Follow-up minus Baseline | 4 | -3.5 (-6.8, 7.2) | 3 | -9.0 (-14.3, -0.7) | 4 | 0.8 (-2.7, 3.7) | 0.17 |
| P-value: Follow-up vs. Baseline |  | 0.63 |  | 0.18 |  | 0.69 |  |
|  |  |  |  |  |  |  |  |
| **Orthostatic heart rate (HR)** |  |  |  |  |  |  |  |
| 0 minutes |  |  |  |  |  |  |  |
| Baseline | 4 | 87.0 (68.0, 107.0) | 3 | 74.0 (67.0, 80.0) | 4 | 67.0 (54.0, 98.0) |  |
| Follow-up | 4 | 77.0 (70.0, 94.0) | 3 | 62.0 (60.0, 78.0) | 4 | 69.5 (62.0, 91.0) |  |
| Follow-up minus Baseline | 4 | -8.0 (-17.0, 2.0) | 3 | -5.0 (-14.0, -2.0) | 4 | 2.0 (-7.0, 9.0) | 0.26 |
| P-value: Follow-up vs. Baseline |  | 0.18 |  | 0.19 |  | 0.74 |  |
| 3 minutes |  |  |  |  |  |  |  |
| Baseline | 4 | 86.0 (66.0, 94.0) | 3 | 73.0 (70.0, 97.0) | 4 | 74.5 (70.0, 97.0) |  |
| Follow-up | 4 | 82.0 (75.0, 90.0) | 3 | 66.0 (60.0, 83.0) | 4 | 68.5 (59.0, 90.0) |  |
| Follow-up minus Baseline | 4 | -1.5 (-9.0, 9.0) | 3 | -13.0 (-14.0, -4.0) | 4 | -3.0 (-7.0, 7.0) | 0.18 |
| P-value: Follow-up vs. Baseline |  | 0.86 |  | 0.083 |  | 0.65 |  |
| 6 minutes |  |  |  |  |  |  |  |
| Baseline | 4 | 88.5 (68.0, 106.0) | 3 | 73.0 (64.0, 81.0) | 4 | 71.0 (54.0, 101.0) |  |
| Follow-up | 4 | 86.0 (78.0, 97.0) | 3 | 66.0 (60.0, 87.0) | 4 | 69.0 (63.0, 89.0) |  |
| Follow-up minus Baseline | 4 | -0.5 (-13.0, 10.0) | 3 | 2.0 (-13.0, 6.0) | 4 | -2.0 (-12.0, 9.0) | 0.99 |
| P-value: Follow-up vs. Baseline |  | 0.88 |  | 0.80 |  | 0.72 |  |
| 9 minutes |  |  |  |  |  |  |  |
| Baseline | 4 | 92.5 (66.0, 101.0) | 3 | 74.0 (63.0, 84.0) | 4 | 71.5 (58.0, 102.0) |  |
| Follow-up | 4 | 84.5 (78.0, 98.0) | 3 | 71.0 (64.0, 87.0) | 4 | 70.5 (58.0, 95.0) |  |
| Follow-up minus Baseline | 4 | -2.5 (-15.0, 13.0) | 3 | 3.0 (-10.0, 8.0) | 4 | -5.0 (-8.0, 9.0) | 0.95 |
| P-value: Follow-up vs. Baseline |  | 0.81 |  | 0.96 |  | 0.60 |  |
| 12 minutes |  |  |  |  |  |  |  |
| Baseline | 4 | 91.0 (68.0, 108.0) | 3 | 73.0 (64.0, 80.0) | 4 | 79.0 (59.0, 98.0) |  |
| Follow-up | 4 | 90.5 (79.0, 99.0) | 3 | 69.0 (58.0, 86.0) | 4 | 72.0 (59.0, 89.0) |  |
| Follow-up minus Baseline | 4 | 1.0 (-12.0, 11.0) | 3 | 5.0 (-15.0, 6.0) | 4 | -4.0 (-21.0, 6.0) | 0.75 |
| P-value: Follow-up vs. Baseline |  | 0.97 |  | 0.86 |  | 0.41 |  |
| 15 minutes |  |  |  |  |  |  |  |
| Baseline | 4 | 88.0 (75.0, 103.0) | 3 | 76.0 (64.0, 87.0) | 4 | 69.0 (59.0, 100.0) |  |
| Follow-up | 4 | 87.5 (77.0, 103.0) | 3 | 63.0 (62.0, 93.0) | 4 | 69.5 (58.0, 87.0) |  |
| Follow-up minus Baseline | 4 | -1.5 (-9.0, 13.0) | 3 | -1.0 (-14.0, 6.0) | 4 | -3.0 (-13.0, 6.0) | 0.84 |
| P-value: Follow-up vs. Baseline |  | 0.96 |  | 0.66 |  | 0.47 |  |
| Mean of 0 to 15 minutes |  |  |  |  |  |  |  |
| Baseline | 4 | 88.8 (68.5, 103.2) | 3 | 73.8 (65.3, 84.8) | 4 | 71.0 (57.0, 99.3) |  |
| Follow-up | 4 | 85.5 (76.3, 94.8) | 3 | 66.2 (60.7, 85.7) | 4 | 69.8 (59.8, 90.2) |  |
| Follow-up minus Baseline | 4 | -2.3 (-10.3, 7.8) | 3 | 0.8 (-13.2, 0.8) | 4 | -3.6 (-9.2, 7.7) | 0.95 |
| P-value: Follow-up vs. Baseline |  | 0.74 |  | 0.50 |  | 0.58 |  |
|  |  |  |  |  |  |  |  |
| **Orthostatic MAP** |  |  |  |  |  |  |  |
| 0 minutes |  |  |  |  |  |  |  |
| Baseline | 4 | 96.7 (79.3, 103.7) | 3 | 99.3 (98.3, 113.7) | 4 | 103.3 (93.7, 115.7) |  |
| Follow-up | 4 | 92.2 (84.7, 101.7) | 3 | 102.3 (82.3, 103.7) | 4 | 100.0 (96.3, 119.7) |  |
| Follow-up minus Baseline | 4 | -4.5 (-19.0, 22.3) | 3 | -10.0 (-16.0, 3.0) | 4 | 1.3 (-6.7, 4.0) | 0.69 |
| P-value: Follow-up vs. Baseline |  | 0.88 |  | 0.30 |  | 1.00 |  |
| 3 minutes |  |  |  |  |  |  |  |
| Baseline | 4 | 91.8 (71.3, 108.0) | 3 | 112.3 (111.0, 113.7) | 4 | 98.2 (94.3, 131.0) |  |
| Follow-up | 4 | 96.5 (82.3, 101.0) | 3 | 104.0 (80.3, 106.3) | 4 | 99.2 (94.7, 120.7) |  |
| Follow-up minus Baseline | 4 | -0.2 (-9.7, 23.3) | 3 | -8.3 (-30.7, -7.3) | 4 | -0.2 (-10.3, 2.7) | 0.15 |
| P-value: Follow-up vs. Baseline |  | 0.67 |  | 0.18 |  | 0.55 |  |
| 6 minutes |  |  |  |  |  |  |  |
| Baseline | 4 | 97.3 (84.0, 103.3) | 3 | 106.7 (96.7, 113.3) | 4 | 97.3 (96.3, 131.0) |  |
| Follow-up | 4 | 91.8 (79.0, 94.3) | 3 | 98.0 (85.0, 104.0) | 4 | 98.8 (95.3, 124.3) |  |
| Follow-up minus Baseline | 4 | -7.3 (-11.3, 1.0) | 3 | -9.3 (-21.7, 1.3) | 4 | -1.2 (-6.7, 4.3) | 0.33 |
| P-value: Follow-up vs. Baseline |  | 0.11 |  | 0.28 |  | 0.65 |  |
| 9 minutes |  |  |  |  |  |  |  |
| Baseline | 4 | 96.2 (90.7, 109.0) | 3 | 108.3 (103.3, 114.0) | 4 | 95.8 (94.0, 113.0) |  |
| Follow-up | 4 | 90.7 (83.3, 97.7) | 3 | 97.3 (85.3, 106.0) | 4 | 99.7 (89.7, 108.0) |  |
| Follow-up minus Baseline | 4 | -6.8 (-11.3, -4.7) | 3 | -8.0 (-23.0, -6.0) | 4 | -1.2 (-5.0, 5.7) | 0.074 |
| P-value: Follow-up vs. Baseline |  | 0.014 |  | 0.15 |  | 0.88 |  |
| 12 minutes |  |  |  |  |  |  |  |
| Baseline | 4 | 99.0 (85.7, 108.0) | 3 | 100.7 (90.3, 120.0) | 4 | 98.3 (93.7, 110.0) |  |
| Follow-up | 4 | 98.8 (77.3, 107.0) | 3 | 90.3 (80.7, 104.3) | 4 | 95.5 (94.3, 107.7) |  |
| Follow-up minus Baseline | 4 | -4.5 (-22.0, 21.3) | 3 | -15.7 (-20.0, 0.0) | 4 | -2.3 (-3.3, 0.7) | 0.54 |
| P-value: Follow-up vs. Baseline |  | 0.81 |  | 0.19 |  | 0.12 |  |
| 15 minutes |  |  |  |  |  |  |  |
| Baseline | 4 | 94.2 (86.3, 106.0) | 3 | 103.7 (98.7, 116.3) | 4 | 93.5 (92.7, 120.0) |  |
| Follow-up | 4 | 93.5 (88.7, 99.7) | 3 | 96.7 (82.3, 101.7) | 4 | 103.2 (96.3, 115.7) |  |
| Follow-up minus Baseline | 4 | -0.7 (-6.3, 2.3) | 3 | -14.7 (-21.3, -2.0) | 4 | 4.0 (-4.3, 15.0) | 0.042 |
| P-value: Follow-up vs. Baseline |  | 0.56 |  | 0.16 |  | 0.32 |  |
| Mean of 0 to 15 minutes |  |  |  |  |  |  |  |
| Mean of 0 to 15 minutes |  |  |  |  |  |  |  |
| Baseline | 4 | 93.8 (86.9, 106.3) | 3 | 104.8 (100.1, 115.2) | 4 | 97.3 (94.9, 120.1) |  |
| Follow-up | 4 | 94.9 (85.4, 95.4) | 3 | 98.1 (82.7, 104.3) | 4 | 99.3 (94.7, 116.0) |  |
| Follow-up minus Baseline | 4 | -2.8 (-10.9, 6.2) | 3 | -10.8 (-22.1, -2.0) | 4 | 0.1 (-4.1, 3.3) | 0.14 |
| P-value: Follow-up vs. Baseline |  | 0.52 |  | 0.18 |  | 0.94 |  |
|  |  |  |  |  |  |  |  |
| **Supine MAP** |  |  |  |  |  |  |  |
| 0 minutes |  |  |  |  |  |  |  |
| Baseline | 4 | 95.2 (90.0, 102.3) | 3 | 111.7 (105.3, 116.0) | 4 | 93.8 (87.7, 105.7) |  |
| Follow-up | 4 | 97.8 (95.7, 101.0) | 3 | 99.7 (90.3, 103.3) | 4 | 93.2 (82.7, 107.7) |  |
| Follow-up minus Baseline | 4 | 1.8 (-3.7, 9.7) | 3 | -8.3 (-25.7, -5.7) | 4 | -0.2 (-10.7, 6.7) | 0.084 |
| P-value: Follow-up vs. Baseline |  | 0.50 |  | 0.17 |  | 0.79 |  |
| 3 minutes |  |  |  |  |  |  |  |
| Baseline | 4 | 96.5 (86.3, 106.3) | 3 | 109.3 (103.0, 110.7) | 4 | 91.3 (90.0, 109.0) |  |
| Follow-up | 4 | 96.8 (89.3, 98.0) | 3 | 99.0 (90.0, 104.3) | 4 | 93.0 (84.3, 102.7) |  |
| Follow-up minus Baseline | 4 | 1.3 (-17.0, 9.7) | 3 | -5.0 (-20.7, -4.0) | 4 | -4.0 (-7.7, 7.0) | 0.48 |
| P-value: Follow-up vs. Baseline |  | 0.86 |  | 0.21 |  | 0.56 |  |
| 6 minutes |  |  |  |  |  |  |  |
| Baseline | 4 | 98.8 (76.0, 105.7) | 3 | 105.0 (100.7, 110.3) | 4 | 92.2 (91.3, 103.0) |  |
| Follow-up | 4 | 89.2 (88.7, 97.3) | 3 | 98.7 (90.7, 106.0) | 4 | 93.2 (82.3, 105.3) |  |
| Follow-up minus Baseline | 4 | -5.8 (-16.3, 13.0) | 3 | -2.0 (-19.7, 1.0) | 4 | -0.7 (-10.3, 7.0) | 0.78 |
| P-value: Follow-up vs. Baseline |  | 0.59 |  | 0.40 |  | 0.78 |  |
| Mean of 0 to 6 minutes |  |  |  |  |  |  |  |
| Baseline | 4 | 96.6 (84.6, 104.8) | 3 | 108.7 (103.0, 112.3) | 4 | 92.4 (89.7, 105.9) |  |
| Follow-up | 4 | 94.7 (92.4, 97.3 | 3 | 99.1 (90.3, 104.6) | 4 | 93.1 (83.1, 105.2) |  |
| Follow-up minus Baseline | 4 | -0.9 (-12.3, 10.8) | 3 | -4.1 (-22.0, -3.9) | 4 | -1.6 (-9.6, 6.9) | 0.39 |
| P-value: Follow-up vs. Baseline |  | 0.88 |  | 0.24 |  | 0.69 |  |
| For comparisons of follow-up vs. baseline values within a given treatment group, p-values result from a paired t-test. For comparisons of follow-up minus baseline values between the three treatment groups, p-values result from a one-way ANOVA. | | | | | | | |
